# Development and Validation of Machine Learning‐Based Marker for Early Detection and Prognosis Stratification of Nonalcoholic Fatty Liver Disease

**DOI:** 10.1002/advs.202410527

**Published:** 2025-05-28

**Authors:** Lushan Xiao, Lin Zeng, Jiaren Wang, Chang Hong, Ziyong Zhang, Chengkai Wu, Hao Cui, Yan Li, Ruining Li, Shengxing Liang, Qijie Deng, Wenyuan Li, Xuejing Zou, Pengcheng Ma, Li Liu

**Affiliations:** ^1^ Department of Health Management Nanfang Hospital, Southern Medical University Guangzhou 510515 China; ^2^ Guangdong Provincial Key Laboratory of Viral Hepatitis Research Department of Infectious Diseases Nanfang Hospital, Southern Medical University Guangzhou 510515 China; ^3^ Department of Gastroenterology Shenzhen Hospital Southern Medical University Shenzhen 518133 China; ^4^ School of Public Health Southern Medical University Guangzhou 510515 China; ^5^ School of Health Management Southern Medical University Guangzhou 510515 China; ^6^ Nanfang Hospital, Southern Medical University Guangzhou 510515 China

**Keywords:** nonalcoholic fatty liver disease, machine learning, multimodal, severe liver disease, prognosis, stratification

## Abstract

Nonalcoholic fatty liver disease (NAFLD) is the leading cause of chronic liver disease and is considered the hepatic manifestation of metabolic syndrome, triggering out adverse outcomes. A stacked multimodal machine learning model is constructed and validated for early identification and prognosis stratification of NAFLD by integrating genetic and clinical data sourced from 36 490 UK Biobank and 9 007 Nanfang Hospital participants and extracted its probabilities as in‐silico scores for NAFLD (ISNLD). The efficacy of ISNLD is evaluated for the early prediction of severe liver disease (SeLD) and analyzed its association with metabolism‐related outcomes. The multimodal model performs satisfactorily in classifying individuals into low‐ and high‐risk groups for NAFLD, achieving area under curves (AUCs) of 0.843, 0.840, and 0.872 within training, internal, and external test sets, respectively. Among high‐risk group, ISNLD is significantly associated with intrahepatic and metabolism‐related complications after lifestyle factors adjustment. Further, ISNLD demonstrates notable capability for early prediction of SeLD and further stratifies high‐risk subjects into three risk subgroups of elevated risk for adverse outcomes. The findings emphasize the model's ability to integrate multimodal features to generate ISNLD, enabling early detection and prognostic prediction of NAFLD. This facilitates personalized stratification for NAFLD and metabolism‐related outcomes based on digital non‐invasive markers, enabling preventive interventions.

## Graphical abstract

This study constructs a stacked multimodal machine learning model for nonalcoholic fattly liver disease (NAFLD) by integrating genetic and clinical features, and synthesize an in‐silico quantitative marker (ISNLD) that enables personalized risk stratification for intrahepatic and extrahepatic outcomes of high‐risk individuals of NAFLD. The novel digital score demonstrates potential to enhance screening, guide preventive interventions, and optimize follow‐up management for NAFLD.

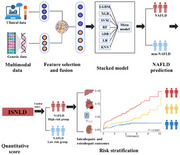

## 1 Introduction

Nonalcoholic fatty liver disease (NAFLD) is a multisystem disease that is considered the hepatic manifestation of the metabolic syndrome.^[^ ^1^ ^]^ Over the past three decades, NAFLD has gradually evolved into the most common chronic liver disease, with its global prevalence rising from 25% to 38%.^[^ ^2^, ^3^ ^]^ A review found a staggering increase in the prevalence of NAFLD in China, accounting for nearly 30% of the total population,^[^ ^4^ ^]^ surpassing those of European countries and the United States^[^ ^5^ ^]^ and making it the leading cause of chronic liver disease in China. Owing to its high prevalence, NAFLD has emerged as the primary driver behind the rapidly increasing liver‐related mortality worldwide. Furthermore, it is gradually becoming a significant cause of end‐stage liver disease and liver transplantation.^[^ ^6^ ^]^ Additionally, multiple studies have substantiated the close association between NAFLD and metabolism‐related adverse events,^[^ ^7^ ^]^ including cardiovascular disease,^[^ ^8^ ^]^ type 2 diabetes mellitus (DmT2),^[^ ^9^ ^]^ and chronic kidney disease (CKD).^[^ ^10^ ^]^ Despite the heavy disease burden, approved pharmaceutical treatments for NAFLD are scarce.^[^ ^11^ ^]^ Hence, the prompt identification and prognostic stratification of NAFLD would aid in disease prevention and personalized intervention.

Although liver biopsy is considered the gold standard for diagnosing and assessing the severity of NAFLD, its widespread use is limited due to its invasiveness, high cost, scalability constraints, and patient reluctance.^[^ ^12^ ^]^ Non‐invasive tests (NIT) offer a convenient and cost‐effective means of disease risk assessment for NAFLD.^[^ ^13^ ^]^ Several NITs derived from serum biomarkers, including Enhanced Liver Fibrosis (ELF) score, Fibrosis‐4 Index (FIB‐4), and NAFLD Fibrosis Score (NFS), are used for diagnosing and assessing NAFLD.^[^ ^14^ ^]^ Although these models exhibit promise for clinical use, they require enhanced accuracy^[^ ^15^ ^]^ and depend predominantly on clinical indicators. NAFLD is a complex disease that exhibits substantial variability during progression among participants. The interaction between environmental factors and the polygenic genetic background of susceptible hosts can affect the disease phenotype and progression.^[^ ^16^ ^]^ *PNPLA3* has been identified as a major common genetic determinant of NAFLD, and *TM6SF2*, *MBOAT7*, and *GCKR* have been shown to contribute significantly.^[^ ^17^ ^]^ Combining clinical and genetic indicators may be an effective way to improve NIT efficiency.

Machine learning (ML) is an effective option when datasets are too large (many individual data points) or too complex (containing a large number of features) for manual analysis.^[^ ^18^ ^]^ ML has been applied to develop diagnostic scores for various diseases, including systemic lupus erythematosus,^[^ ^19^ ^]^ Alzheimer's disease,^[^ ^20^ ^]^ and coronary artery disease (CAD).^[^ ^21^ ^]^ Compared with traditional NITs for NAFLD, ML models perform better in terms of sensitivity and specificity.^[^ ^22^, ^23^ ^]^ Modeling based on multimodal data can reduce the inherent biases of using single‐modal data and improve the accuracy and generalization ability of the model.^[^ ^24^, ^25^ ^]^ However, the ability of ML models to integrate clinical and genetic features to predict the occurrence and progression of NAFLD requires further exploration. Moreover, existing NIT models primarily serve as classification tools for identifying NAFLD, but lack the ability to quantitatively assess the disease on a continuous scale. Quantitative assessment of NAFLD and its related disease risks is conducive to the dynamic observation of disease conditions.

In the present multicenter study, we aimed to develop and validate a stacked multimodal ML model incorporating genetic and clinical data and synthesize an in‐silico quantitative marker for NAFLD (ISNLD), which can support the prompt identification of NAFLD and facilitate early risk stratification of severe liver disease (SeLD) and metabolism‐related outcomes.

## 2 Results

### 2.1 Study Workflow

**Figure 1:**
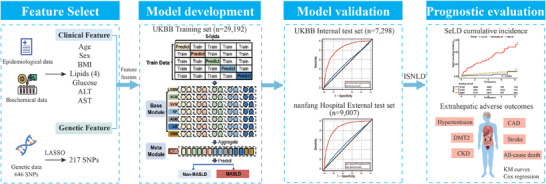
Workflow of the established stacked model and study design. SNP, single nucleotide polymorphism; BMI, Body mass index; ALT, alanine aminotransferase; AST, aspartate aminotransferase; LASSO, least absolute shrinkage, and selection operator; LGBM, Light Gradient Boosting Machine; XGB, classical extreme gradient boosting; SVM, Support Vector Machine; RF, Random Forest; ADB, Adaptive Boosting; LR, Logistic regression; KNN, K‐Nearest Neighbour. AUC, the area under the receiver operating characteristic curve; ISNLD, probabilities as in‐silico quantitative marker for NAFLD; SeLD, severe liver disease; DmT2, type 2 diabetes mellitus; CKD, chronic kidney disease; CAD, coronary artery disease.

The overall study design is shown in **Figure** **1**. This study consisted of two main components. The first part used genetic and clinical data from the UK Biobank cohort (UKBB, 36490 participants) to train and internally test a stacked multimodal ML model to accurately predict NAFLD. Subsequently, to ensure the generalizability of the model, we validated it using an external test set (health examinee dataset from Nanfang Hospital, 9007 participants). In the second part, we extracted probability scores from the multimodal model to construct ISNLD as a non‐invasive quantitative marker for NAFLD, intended for the prognostic prediction of future risks associated with intrahepatic and extrahepatic adverse outcomes (Figure S1, Supporting Information). ISNLD was used to classify participants into low‐ and high‐risk groups for NAFLD based on the maximum value of the Youden index. Further analysis in the high‐risk group, we evaluated the efficacy of ISNLD in predicting the risk of SeLD, and explored its association with metabolism‐related adverse outcomes.

### 2.2 Clinical Features of the Two Data Sets

A total of 36 490 participants from the UKBB and 9007 from Nanfang Hospital were included as the development and test cohorts for analysis, respectively (**Table** **1**). The baseline characteristics of the participants from the UKBB and Nanfang Hospital differed. Participants from Nanfang Hospital had a lower prevalence of NAFLD (24.87% vs 32.35%; *p* < 0.001). The average age of participants in the UKBB cohort was greater than that of participants in the Nanfang Hospital cohort (56 vs 36 years; *p* < 0.001), because the participants in the health examinee dataset were largely working people whose overall age was young. Participants in the UKBB cohort had a higher body mass index (BMI) and higher triglycerides (TG), cholesterol (CHO), low‐density lipoprotein‐cholesterol (LDL‐C), high‐density lipoprotein‐cholesterol (HDL‐C), blood glucose (Glu), alanine aminotransferase (ALT), and aspartate aminotransferase (AST) level*s* (*p* < 0.001). The characteristics of the study participants in the training and internal test sets of the UKBB cohort are shown in Table S1 (Supporting Information). The training and internal test sets were almost balanced with respect to baseline characteristics.

**Table 1:** Comparison of clinical features between two cohorts.

Characteristic	UK Biobank [*n* = 36 490]	Nanfang Hospital [*n* = 9007]	*p*‐value
Age, years	56 (49, 61)	36 (30, 45)	<0.001
Sex, n (%)			0.738
Female	19 526 (53.51)	4838 (53.71)	
Male	16 964 (46.49)	4169 (46.29)	
BMI, kg/m^2^	26.26 (23.76, 29.36)	22.63 (20.48, 25.06)	<0.001
TG, mmol/L	1.42 (1, 2.08)	1.09 (0.79, 1.62)	<0.001
CHO, mmol/L	5.63 (4.93, 6.38)	5.02 (4.4, 5.7)	<0.001
LDL‐C, mmol/L	3.51 (2.96, 4.1)	3.11 (2.65, 3.63)	<0.001
HDL‐C, mmol/L	1.41 (1.18, 1.68)	1.36 (1.18, 1.58)	<0.001
Glu, mmol/L	4.89 (4.57, 5.25)	4.77 (4.5, 5.08)	<0.001
ALT, U/L	20.01 (15.25, 27.63)	16 (11, 25)	<0.001
AST, U/L	24.3 (20.9, 28.9)	18 (15, 22)	<0.001
NAFLD, n (%)	11 803 (32.35)	2240 (24.87)	<0.001

For continuous features median (interquartile range) is reported. For categorical features, count (%) is reported. Continuous variables were assessed using the Mann–Whitney *U* test. Categorical variables were evaluated using chi‐square or Fisher's exact tests; *p*‐value is used to assess the statistical significance of clinical variables between the UK Biobank and Nanfang Hospital cohorts. BMI, Body mass index; TG, Triglyceride; CHO, Cholesterol; LDL‐C, Low‐density lipoprotein‐cholesterol; HDL‐C, High‐density lipoprotein‐cholesterol; Glu, blood glucose; ALT, alanine aminotransferase; AST, aspartate aminotransferase; NAFLD, Nonalcoholic fatty liver disease.

### 2.3 Associations of SNPs with NAFLD in the UKBB Cohort

**Figure 2:**
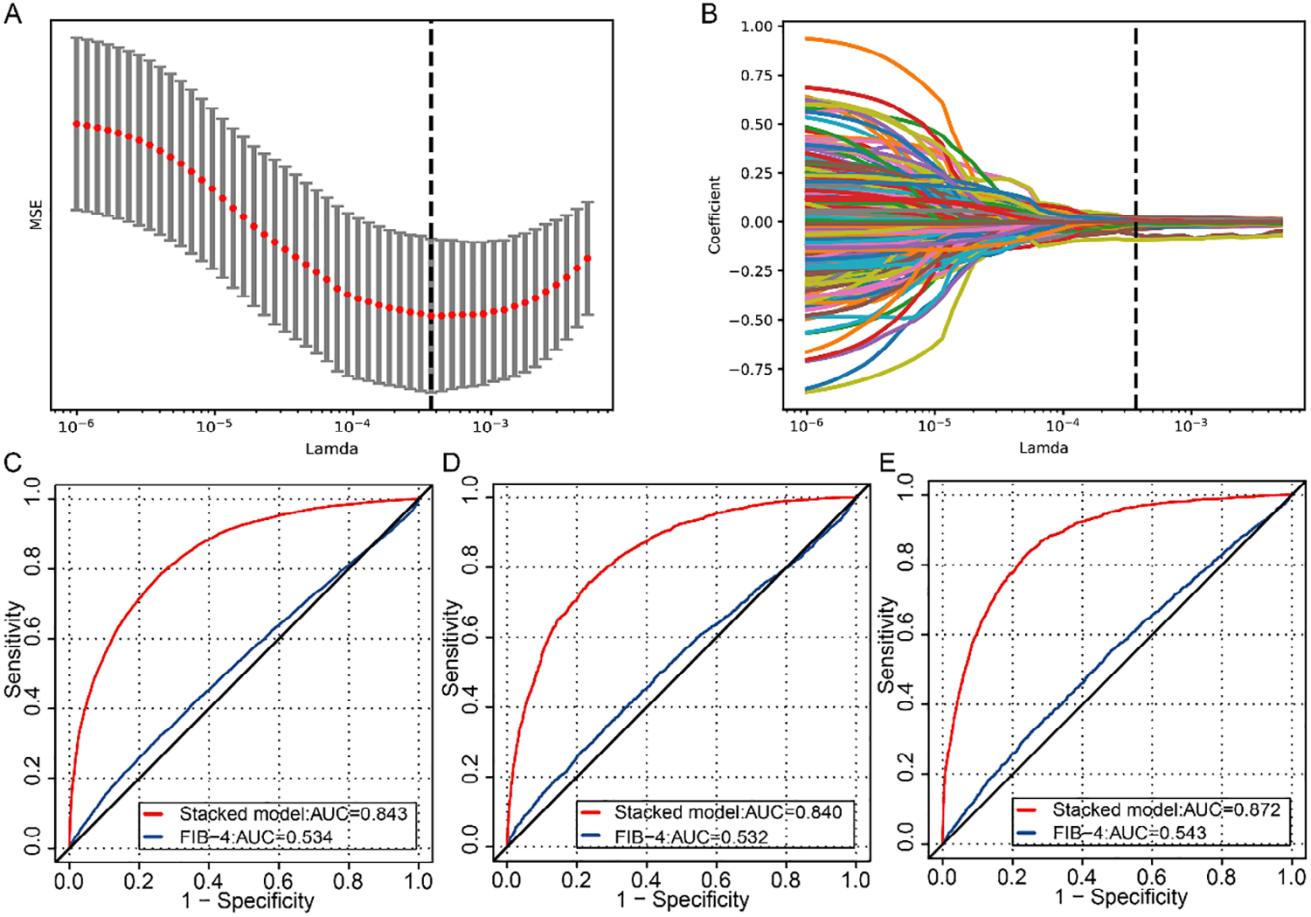
Performance of the stacked multimodal ML model for predicting NAFLD. A) Selection of SNP through LASSO in the training set; A dotted vertical line is drawn at the optimal lambda values by minimum criteria, which is 217; The lambda.min means the lambda at which the minimal MSE is achieved through 5‐fold cross‐validation; B) LASSO coefficient profiles of SNPs; C) the ROC analyses for predicting NAFLD in the training test set with the stacked ML model and FIB‐4; D) the ROC analyses for predicting NAFLD in the internal test set with the stacked ML model and FIB‐4; E) the ROC analyses for predicting NAFLD in the external test set with the stacked ML model and FIB‐4. ML, machine learning; NAFLD, non‐alcoholic fatty liver disease; MSE, mean square error; SNP, single nucleotide polymorphism; LASSO, least absolute shrinkage and selection operator; ROC, receiver operator characteristic.

We used the least absolute shrinkage and selection operator (LASSO) to select the most valuable genetic variables (lambda. min) to identify NAFLD from 646 single nucleotide polymorphisms (SNPs) in the GWAS analyses in the UKBB or in previous studies. The lambda. min indicated the lambda value at which the minimal mean square error (MSE) was achieved through 5‐fold cross‐validation (**Figure** **2**A,B). In the training set, 217 SNPs were selected as significantly (5 × 10^−6^) associated with NAFLD. A total of 52 genes could be identified after functional annotation, including PNPLA3, SUGP1, TM6SF2, LEPR, APOE, GCKR, and SAMM50. Their lead SNPs' effects and *P*‐values are presented in Table S2 (Supporting Information).

**Figure 3:**
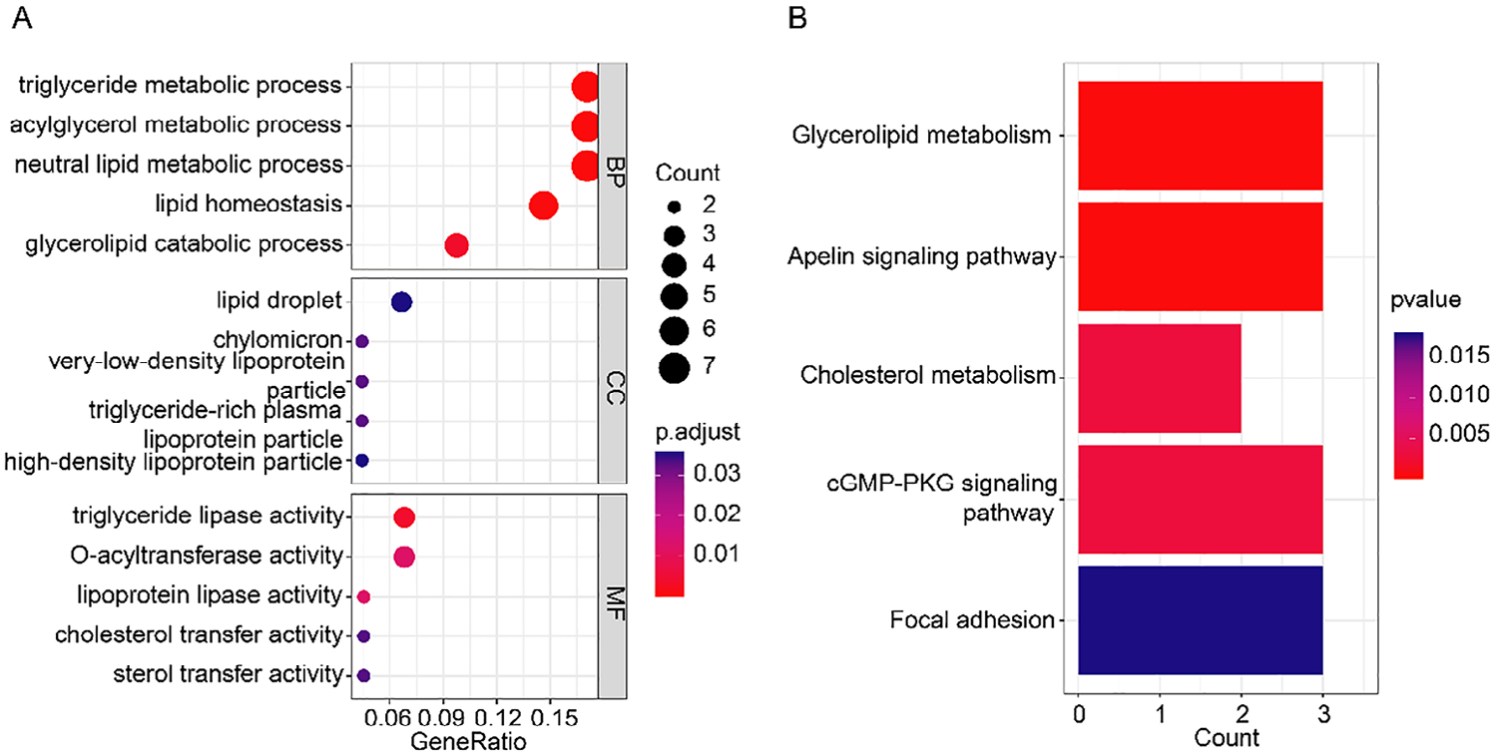
Enrichment analysis of gene set of selected genetic features. A) Top five enriched items for BP, CC, and MF, respectively in the GO analysis. B) Enrichment ratio of five significant pathways in the KEGG analysis. GO, Gene Ontology; KEGG, Kyoto Encyclopedia of Genes and Genomes; BP, Biological Process; CC, Cellular Component; MF, Molecular Function.

Next, to gain insight into the association between the selected genetic features and NAFLD, the enrichment analysis was conducted by using the annotated gene sets. Gene Ontology (GO) analysis was performed at three levels: biological process (BP), cellular component (CC), and molecular function (MF), and the enriched items included: triglyceride metabolic process, lipid droplet, and triglyceride lipase activity (**Figure** **3**A). Kyoto Encyclopedia of Genes and Genomes (KEGG) analysis showed that glycerolipid metabolism, apelin signaling pathway, cholesterol metabolism, cGMP‐PKG signaling pathway, and focal adhesion were significantly enriched (Figure 3B). The results indicated that the enriched functions and signaling pathways were mainly related to lipid metabolism and transport.

### 2.4 Performance of the Stacked ML Model in the Training Set

In total, 217 genetic and 10 clinical features were selected as predictors for NAFLD. The seven base classifiers in the base module were used to fuse the input clinical and genetic features in the training set to predict NAFLD. Logistic regression (LR) had the best comprehensive performance during the five‐fold cross‐validation procedure. The area under curve (AUC) and exact values in the training set for the single classifiers were presented in Table S3 (Supporting Information).

Subsequently, based on the prediction results of these seven classifiers in the five‐fold cross‐validation, we constructed the stacked model through the LR classifier in the fusion module and compared it with FIB‐4. The AUC of the stacked multimodal model for predicting NAFLD was 0.843 (95% confidence interval (CI) 0.838, 0.848) in the training set (Figure 2C). In comparison, the AUC of FIB‐4 was 0.534 (95% CI 0.527, 0.541). The stacked model performed better than the individual classifiers. Our results suggest that ML models incorporating clinical and genetic features have superior efficacy in predicting NAFLD. Additionally, decision curve analyses graphically demonstrated that the ML model provided a larger net benefit across the range of reasonable threshold probabilities in all data sets (Figure S2A–C, Supporting Information). Moreover, the calibration curve exhibited good consistency between the predicted NAFLD probability of the ML model and the actual probability (Figure S2D–F, Supporting Information).

Moreover, we displayed the top 20 features of six base classifiers (Figure S3, Supporting Information). Clinical features, including BMI, TG, and ALT, were emphasized across all classifiers. Additionally, significant contributions were observed from certain SNPs located predominantly in genes such as PNPLA3, TM6SF2, and GCKR, which mainly play a role in liver fat accumulation and are associated with an elevated risk of NAFLD.

### 2.5 Performance of the Stacked ML Model in the Internal and External Test Sets

We further evaluated the performance of the stacked models on the internal and external test sets. The AUCs of the stacked model for predicting NAFLD were 0.840 (95% CI 0.831, 0.850) and 0.872 (95% CI 0.864, 0.880) in the internal and external test sets, respectively (Figure 2D,E). The performance of the stacked model was significantly better than that of the FIB‐4 index[internal test set: 0.532 (95% CI 0.518, 0.547), external test set: 0.543 (95% CI 0.530, 0.557)].

### 2.6 Characteristics of the ISNLD Score

We utilized the NAFLD probabilities derived from the stacked multimodal model to generate ISNLD for the participants and assessed its prognostic value. In the training, internal test, and external test sets, ISNLD showed a superior ability to identify NAFLD. ISNLD in the NAFLD group was significantly increased compared to the control group (training set 0.214 vs 0.519, *p* < 0.05; internal test set 0.214 vs 0.543, *p* < 0.05; external test set 0.163 versus 0.463, *p* < 0.05) (Figure S4, Supporting Information). The risk factors for NAFLD were significantly associated with ISNLD in all three data sets (Figures S5–S7, Supporting Information). In the training set, ISNLD increased monotonically by 0.035 (95% CI 0.031, 0.039; *p* < 0.05) per decade of age. Participants with male sex[0.085 (0.079, 0.091); *p* < 0.05], obesity[0.434 (0.428, 0.440); *p* < 0.05], dyslipidemia[0.160 (0.154, 0.166); *p* < 0.05] and dysglycemia[0.403 (0.369, 0.438); *p* < 0.05] had a higher ISNLD than those without these factors. Higher AST[0.139 (0.137, 0.142), *p* < 0.05] and ALT[0.085(0.082, 0.087), *p* < 0.05] levels were associated with greater ISNLD. ISNLD captured the axes of NAFLD risk from the SNP score; ISNLD increased by 0.070 (95% CI: 0.067, 0.073; *p* < 0.05) per quartile increase in SNP score. For each quartile increase in magnetic resonance imaging‐proton density fat fraction (MRI‐PDFF), ISNLD increased by 0.110 (95% CI: 0.108, 0.112; *p* < 0.05), suggesting that ISNLD was a potential indicator of the severity of NAFLD steatosis. The association between ISNLD and these factors showed a similar trend in both internal and external test sets. These results further underscore the potential of ISNLD as an effective non‐invasive biomarker for NAFLD.

### 2.7 Prognostic Evaluation of ISNLD

NAFLD is closely associated with both intrahepatic and extrahepatic adverse outcomes, imposing a significant global disease burden. Given this substantial clinical impact, we assessed whether ISNLD could serve as a quantitative biomarker for risk stratification of SeLD and metabolism‐related outcomes. According to the maximum Youden index of ISNLD for detecting NAFLD (0.252), the population was divided into low‐ and high‐risk groups for NAFLD. Baseline characteristics differed between the two groups across the training, internal test, and external sets (Tables S4–S6, Supporting Information).

**Figure 4:**
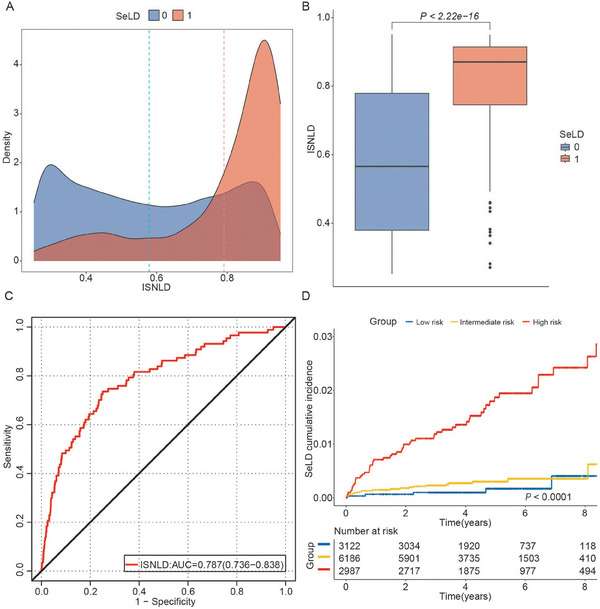
Performance of ISNLD for predicting SeLD in the high‐risk group for NAFLD of training set. A) Density plots of the ISNLD score between normal (blue) and SeLD (red) groups. B) Comparison of ISNLD percentile between normal (blue) and SeLD (red) group, *p* < 2.22e‐16. C) The ROC analyses for ISNLD predicting SeLD in the NAFLD high‐risk population. D) The cumulative risks of developing incident SeLD by ISNLD groups, *p* < 0.0001. ISNLD, in‐silico quantitative marker for NAFLD; SeLD, severe liver disease; ROC, receiver operator characteristic.

During a median follow‐up of 4.36 years, SeLD occurred in 97 and 19 participants in the training and internal test sets, respectively. In the training set, ISNLD significantly distinguished between participants with and without SeLD (**Figure** **4**A,B). And the ISNLD demonstrated strong discriminative capacity for predicting SeLD in the NAFLD high‐risk population, achieving an AUC of 0.787 (95% CI: 0.752, 0.822) (Figure 4C). We further stratified NAFLD high‐risk individuals into three clinically meaningful risk subgroups based on ISNLD quartile ranges: low risk (first quartile), intermediate risk (second and third quartiles), and high risk cohorts (fourth quartile). The cumulative incidence curves showed increases in SeLD risk across ascending ISNLD risk subgroups (Figure 4D). The internal test set showed similar results(Figure S8, Supporting Information).

**Figure 5:**
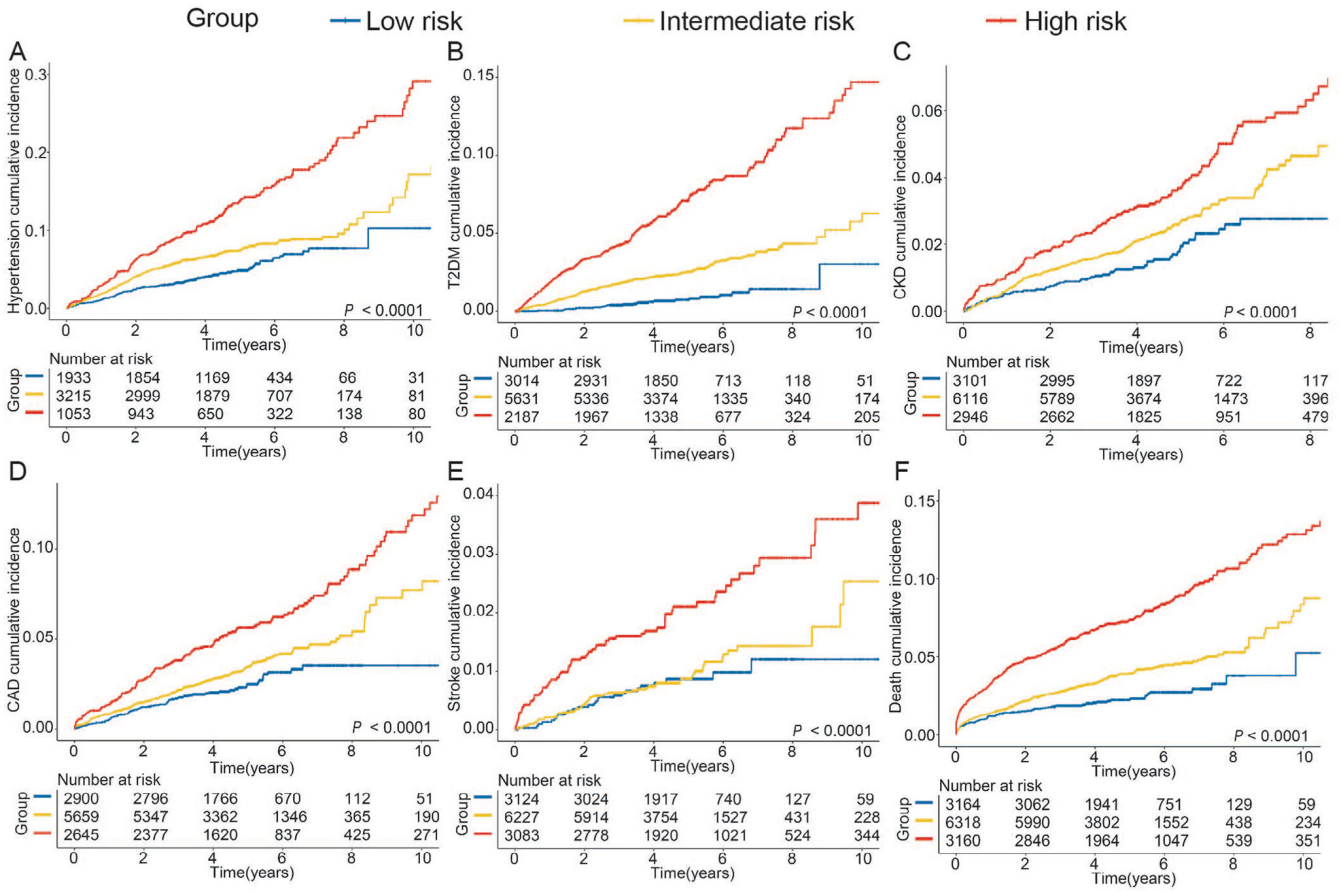
The cumulative incidences of adverse outcomes among the high‐risk group for NAFLD in the training set, by ISNLD groups. A) hypertension; B) DmT2; C) CKD; D) CAD; E) Stroke; F) all‐cause death; the low‐risk group was set as the reference group. ISNLD, in‐silico quantitative marker for NAFLD; DmT2, type 2 diabetes mellitus; CKD, chronic kidney disease; CAD, coronary artery disease.

Additionally, we assessed the association between ISNLD and metabolism‐related outcomes in the training and internal test sets. In the training set, we identified 889, 423, 526, 683, 369, 613, 259, and 902 incident events of hypertension, DmT2, CKD, CAD, heart failure (HF), atrial fibrillation/atrial flutter (AF), stroke, and all‐cause death, respectively (Table S1, Supporting Information). ISNLD was associated with highten risk of metabolism‐related outcomes (Table S7, Supporting Information). The cumulative incidence analysis showed a significant stratification of metabolic risk, with the high‐risk subgroup exhibiting the highest probability of developing metabolism‐related outcomes, while the low‐risk group showed the most favorable prognosis (**Figure** **5**). After adjusting for lifestyle, the hazard ratios (HRs) for the occurrence of hypertension, DmT2, CKD, CAD, sroke, and all‐cause death in high risk group were 2.45[95% CI 1.81, 3.32; *p* < 0.001], 8.93 (95% CI 5.06, 15.74; *p* < 0.001), 2.08 (95% CI 1.38, 3.14; *p* < 0.001), 2.19 (95% CI 1.57, 3.06; *p* < 0.001), 1.72 (95% CI 1.00, 2.95; *p* < 0.001) and 3.42 (95% CI 2.46, 4.77; *p* < 0.001), respectively (Table S8, Supporting Information). Similar results were observed for the internal test set (Figure S9 and Tables S9,S10, Supporting Information).

## 3 Discussion

This study used a large‐scale UKBB cohort as the training and internal test sets and a health examinee dataset from Nanfang Hospital as the external test set to construct a novel stacked multimodal ML model to synthesize an in‐silico quantitative marker for NAFLD (ISNLD). The performance of the proposed model surpassed that of the individual ML models and FIB‐4, demonstrating consistent and satisfactory efficacy. The ISNLD marker enables classifying individuals into low‐ and high‐risk groups for NAFLD, further facilitating stratification into three risk subgroups for SeLD and metabolism‐related outcomes. ISNLD facilitates the early‐stage quantification of SeLD and metabolism‐related outcomes risk, offering robust support for the early screening and stratified management of NAFLD.

According to guidelines from the US Food and Drug Administration, non‐invasive biomarkers are of significant importance in diagnosing clinically relevant.^[^ ^26^ ^]^ Non‐invasive biomarkers are increasingly pivotal in forecasting the prognosis of patients with NAFLD, potentially catalyzing a shift in the NAFLD treatment paradigm from the mere diagnosis of liver pathology to prognosis prediction.^[^ ^12^, ^27^, ^28^, ^29^, ^30^ ^]^ Most existing prognostic models for NAFLD use only clinical features or polygenic risk scores, and are constructed by traditional regression methods. A previous study showed that FIB‐4 combined with instantaneous elastography could stratify patients with NAFLD based on their risk of liver‐related events ^[^ ^27^ ^]^, but the use of imaging methods still limits its generalizability. Another study found that FIB‐4 predicted all‐cause death in patients with NAFLD with an AUC of 0.72, BARD of 0.62, and APRI of 0.52, whereas FIB‐4 predicted SeLD with an AUC of 0.72, BARD of 0.62, and APRI of 0.69.^[^ ^29^ ^]^

There were previous studies that used multimodal data modeling to predict NAFLD and related outcomes, but the performance of models still needed to be improved. Chen et al. utilized PNPLA3‐rs738409‐GG genotype and polygenic risk score to predict cirrhosis risk in patients with NAFLD, achieving AUC values of 0.78 and 0.73, respectively.^[^ ^31^ ^]^ The combining of the PNPLA3‐rs738409 genotype with diabetes status can identify patients with NAFLD currently considered at uncertain risk of cirrhosis, in a similar way to those currently considered at high risk.^[^ ^32^ ^]^ Another study used multivariate LR to develop a NASH PT scoring system based on PNPLA3 and TM6SF2 genotypes and clinical factors;^[^ ^33^ ^]^ the AUC for diagnosing NASH was 0.787–0.859. To our knowledge, there is currently no research assessing the predictive value of non‐invasive markers for extrahepatic adverse outcomes. The non‐invasive marker ISNLD extracted from our multimodal ML model accurately identified the risk of NAFLD and provided early prediction of SeLD (AUC: training set 0.787; internal test set 0.759). ISNLD could also quantify the risk of extrahepatic metabolism‐related outcomes, thereby facilitating personalized patient management strategies.

In addition, compared with conventional machine learning methods, we constructed a stacked machine learning architecture that included seven base modules and a meta module for enhanced robustness and generalizability. For multimodal data processing, we applied an early fusion strategy to process the fused data using a unified algorithm, simplifying the design of the algorithm and optimization process, and making the system easier to debug and maintain. And the universality or transferability of algorithms is a significant gap in the current field of machine learning predictive analytics that limits its widespread adoption and practical use.^[^ ^34^, ^35^ ^]^ Our stacked multimodal algorithm has been demonstrated to have excellent performance in predicting hyperuricemia and related adverse outcomes.^[^ ^36^ ^]^ This partly reflects the transferability of our algorithm, which can be applied to different diseases.

The ISNLD marker integrates ten clinical characteristics that are closely related to the development and progression of NAFLD, including age,^[^ ^37^ ^]^ sex, BMI,^[^ ^38^ ^]^ lipid‐related parameters,^[^ ^39^, ^40^ ^]^ Glu,^[^ ^41^, ^42^ ^]^ AST, and ALT.^[^ ^43^ ^]^ NAFLD has a complex phenotype, and dynamic interactions between genetic and environmental factors are likely to influence disease susceptibility and progression.^[^ ^16^ ^]^ It has been confirmed that liver fat content is significantly heritable, and it is estimated that 38–100% of the variation in liver fat content and NAFLD is caused by genetic factors.^[^ ^44^ ^]^ The ISNLD‐fused 217 genetic features that were significantly associated with NAFLD as screened by LASSO; some of these were confirmed to be closely related to the occurrence and progression of NAFLD at the mechanistic level. PNPLA3 is the most robust and reproducible genetic variant associated with NAFLD, and its homologous variation in European populations results in a ten‐fold increase in the risk of NAFLD‐associated hepatocellular carcinoma.^[^ ^45^ ^]^ TM6SF2 is involved in the accumulation of TG to apolipoprotein B100 in the very low‐density lipoprotein secretion pathway in hepatocytes and quantitatively regulates lipid synthesis and the number of secreted lipoprotein particles.^[^ ^46^ ^]^ GCKR gene variation may regulate the risk of NAFLD associated with obesity and/or elevated TG levels and is significantly associated with liver fat accumulation and liver enzyme levels.^[^ ^47^ ^]^

Except genetic and clinical characteristics, the integration of metabolomic profiles may hold significant potential for enhancing the identification accuracy of NAFLD. Emerging evidence demonstrates the clinical utility of metabolomic signatures in monitoring disease progression, particularly for tracking hepatic fibrosis advancement and facilitating early diagnosis of hepatocellular carcinoma.^[^ ^48^ ^]^ Emerging materials may also advance the diagnosis and treatment of NAFLD. For instance, researchers have proposed an innovative diagnostic approach integrating dried serum spot sampling with nanoparticle‐enhanced laser desorption/ionization mass spectrometry, enabling precise metabolic diseases diagnosis through enhanced molecular profiling.^[^ ^49^ ^]^ In parallel, Cheng Qian et al. introduced a nanomedicine‐encouraged autophagy promoter that facilitates reversal of hepatic fibrosis progression.^[^ ^50^ ^]^ These integrated strategies demonstrate significant potential for improving diagnostic accuracy and therapeutic efficacy in managing NAFLD.

This study has some limitations. First, our data included a small number of participants diagnosed with NAFLD compared to a large number of participants in the UKBB, as the diagnosis was based on MRI‐PDFF rather than abnormal biochemistry or ultrasound findings. The number of participants whose MRI‐PDFF data were available for analysis was rather small compared to the total number of participants in the UKBB. However, MRI‐PDFF is superior to biomarkers or ultrasound for assessing the degree of hepatic steatosis. Second, we developed a stacked ML model in a European cohort and externally tested it in a Chinese cohort; thus, a dataset shift may exist. Although the performance of the ML model was acceptable in the internal and external test sets, it needs to be further validated in other regions or ethnic populations. Third, we only validated the performance of ISNLD on metabolism‐related outcomes using the UKBB dataset. We hoped to collect detailed follow‐up information from the Nanfang Hospital cohort and validate the efficacy of ISNLD in predicting NAFLD and other clinical outcomes.

## 4 Conclusion

We constructed and validated a reliable and practical stacked multimodal ML model for NAFLD based on two large population‐based cohorts and synthesized an in‐silico quantitative marker for NAFLD. The non‐invasive ISNLD marker can timely identify NAFLD participants and predict SeLD at an early stage, and it is significantly associated with an increased risk of metabolism‐related outcomes. Our proposed model and ISNLD may contribute to both screening the high‐risk population of NAFLD and further personalized risk stratification for intrahepatic and extrahepatic outcomes. Future studies with larger numbers and different ethnic populations need to be performed.

## 5 Experimental Section

### Study Population

This study included two cohorts of participants from the UK and China. The UKBB is an ongoing prospective cohort study with clinical and genotypic data, and multiple follow‐ups that enrolled >500 000 participants between 2006 and 2010. After excluding participants who withdrew consent in the UKBB (*n* = 8), participants were excluded with missing covariate or genotype data from the overall study population (*n* = 502381). Finally, 36 490 participants were included and randomly divided in an 8:2 ratio (29192 training set and 7298 internal test set; Figure S10, Supporting Information).

The health examinee dataset of Nanfang Hospital contains information on individuals undergoing health checkups. The data were extracted from individuals aged ≥18 years who visited the hospital between 2015 and 2020 (*n* = 9942). Data collection and preprocessing were performed using the same criteria used in the UKBB, and 9007 participants were included in the external test set (Figure S10, Supporting Information).

### Diagnosis of NAFLD

The diagnostic criteria for NAFLD were based on imaging or liver histological evidence confirming the presence of hepatic steatosis and excluding liver disease due to excessive alcohol consumption or other known causes.^[^ ^51^ ^]^ Due to the lack of liver biopsy data, MRI‐PDFF and International Classification of Diseases (ICD) 9/10 codes (571.5, 571.8, 571.9, K74.0, K74.6, K75.8, and K76.0) were used to identify individuals with NAFLD in the UKBB, and defined MRI‐PDFF ≥ 5% as hepatic steatosis. Additionally, ICD9/10 codes (571.1‐4, 571.6, 572.0, 572.8, 573.3, 573.8‐9, K70.0‐4, K70.9, K71.0‐2, K71.5‐9, K72.0‐1, K72.9, K73.0‐2, K73.8‐9, K74.1‐5, K75.0, K75.2‐4, K75.9, K76.1‐3, K76.6‐9 and K77.0) were used ^[^ ^52^ ^]^ to exclude participants with other liver disease. Participants with MRI‐PDFF <5% and without a diagnosis of NAFLD or other liver diseases based on ICD9/10 codes were selected as healthy controls.

The health examinee dataset of Nanfang Hospital also lacked pathological liver biopsy data, and MRI was not included in routine physical examinations. Liver ultrasonography was used to distinguish between the NAFLD and control groups.

### Clinical Data

In the UKBB, the required clinical data were obtained using the corresponding data‐field code, including age, sex, BMI, Glu, TG, CHO, LDL‐C, HDL‐C, ALT, and AST, and lifestyle factors. Four healthy lifestyle factors: no or moderate alcohol consumption, no smoking, regular physical activity, and a healthy diet were identified (Table S11, Supporting Information).

In the health examinee dataset of Nanfang Hospital, clinical data were obtained from the electronic health record system of physical examinations. Given that most participants underwent continuous health examinations at the Nanfang Hospital Health Management Center, each participant was matched with the results of the biochemical analysis closest to the time the genetic test samples were sent.

### Genotype Data

UKBB genotype data were derived from two independently designed genome‐wide association research chips (genome‐wide association study[GWAS] chips): the Affymetrix UK BiLEVE array and the UK Biobank Axiom array. Genotyping of the health examinee dataset from Nanfang Hospital was performed using the Infinium Chinese Genotyping Array v1.0. Genomic DNA was extracted from the peripheral blood mononuclear cells. Target genetic variants were SNPs that had been either been identified as having a significant association with NAFLD (*p* <5 × 10^−6^)^[^ ^53^ ^]^ in GWAS analyses in the UKBB, or were reported in previous studies (where fatty liver disease was diagnosed by biopsy^[^ ^17^ ^]^ or imaging^[^ ^54^ ^]^) as being associated with NAFLD. The genotype information of these SNPs was extracted as genetic features from the two datasets, and 646 SNPs that exist in both datasets were extracted simultaneously.

### Clinical Outcomes

In the UKBB, detailed follow‐up data of the participants were obtained from their past and future medical and other health‐related records, providing follow‐up information related to cause‐specific mortality and other health events. The prognostic value of ISNLD in predicting both intra‐ and extrahepatic adverse outcomes associated with NAFLD was evaluated. Intrahepatic adverse outcomes refer to SeLD, whereas extrahepatic outcomes were mainly metabolism‐related adverse outcomes, including hypertension, DmT2, CKD, CAD, stroke, HF, AF, and all‐cause death. SeLD was defined as a composite diagnosis of cirrhosis, decompensated liver disease (i.e., esophageal varices with or without bleeding, portal hypertension, hepatorenal syndrome, and liver failure), hepatocellular carcinoma, and/or liver transplantation.^[^ ^55^ ^]^ The identification of these diseases relied on the ICD and self‐reported codes (Table S12, Supporting Information). The time of death and the cause of death were recorded from the National Death Register, and information on deaths in England and Wales was provided from the NHS Data Register. The follow‐up time for each participant was calculated from the baseline diagnosis of NAFLD until the date of identification of any outcome, loss to follow‐up, or last follow‐up (2022‐11‐14), whichever occurred first. Cases occurring before the baseline diagnosis of NAFLD were excluded.

### Feature Selection

Feature selection and normalization were performed on the training set from the UKBB and subsequently applied to both internal and external test sets. This process aimed to optimize the performance and clinical interpretability of the ML model while mitigating its complexity. Ten variables were selected for clinical features based on existing research:^[^ ^56^, ^57^ ^]^ sex, age, BMI, TG, CHO, LDL‐C, HDL‐C, Glu, ALT, and AST. For genetic features, LASSO regression was used to identify the most predictive SNPs associated with NAFLD in the training set (lambda.min). The LASSO regression analysis was executed using “LassoCV” statistical software (Python Foundation). Given the inherent scale variations between genetic and clinical attributes, all selected features were standardized using “StandardScaler” (Python Foundation). Clinical and genetic features were jointly fed into a stacked model for further analysis.

### Model Development and Validation

A stacked multimodal ML architecture with two components, the base module and the meta module, was proposed, which were interconnected in a cascading manner. The base module consists of seven base classifiers that operate independently, including a Light Gradient Boosting Machine (LGBM), classical extreme gradient boosting (XGB), support vector machine (SVM), random forest (RF), adaptive boosting (ADB), logistic regression (LR), and K‐nearest neighbor (KNN), all of which was operated in parallel, independently predict the input features, and subsequently aggregate these predictions. The aggregate results were then transferred to a meta module consisting of a single LR classifier that further processes the output from the base module to derive the final NAFLD prediction.

Throughout the training phase, a 5‐fold cross‐validation method was utilized to bolster model stability and alleviate overfitting. This involved randomly dividing the training set into five distinct subsets of the same size for iterative model training (five iterations in total). Within each iteration, four of the five subsets were concurrently employed for training the seven base classifiers, whereas the remaining subset was used for internal validation purposes. Specifically, the seven base classifiers predicted the outcomes on the remaining subset, enabling an evaluation of the base classifiers’ performance against the ground truth. Simultaneously, these predicted outcomes were aggregated to form the input features for the meta‐classifier. Following the completion of five iterations, an encompassing set of meta‐input features was derived, enabling the training of the meta‐classifier using all features to predict NAFLD. The performance of the stacked model was evaluated using internal and external test sets to assess its efficacy.

### Model Interpretability

It was attempted to explain the model by conducting a feature importance analysis of base classifiers, since a meta‐classifier was trained and made predictions based on the outputs of base classifiers. The individual contributions of features to the predictive capability of the model were identified and ranked by feature importance analysis. This analysis indicated the features with the most influence on the model's predictions, providing insights into the model's decision‐making process and enhancing its interpretability.

### ISNLD Acquisition and Prognostic Evaluation

The stacked multimodal model generated probability scores for each participant, which were used as ISNLD values. The ISNLD ranges from 0 (lowest probability of NAFLD) to 1 (highest probability of NAFLD) and serves as a quantitative marker for NAFLD, predicting future risks associated with SeLD and metabolism‐related outcomes.

In the training set, the ability of ISNLD was evaluated to predict SeLD using receiver operating characteristic (ROC) curve analysis. Participants were then divided into the high‐risk and low‐risk groups for NAFLD by using the maximum value of the Youden index. In the high‐risk group, the participants were further stratified into low risk (first quartile), intermediate risk (second and third quartiles), and high risk subgroups (fourth quartile) based on ISNLD quartiles. The predictive value of ISNLD was assessed in stratifying metabolic risk by evaluating its associations with metabolism‐related outcomes across risk‐stratified subgroups. To validate the discriminatory ability of ISNLD, probability scores were obtained in the internal test set after testing the stacked multimodal model and similarly divided the participants into three groups using quartiles to verify the prognostic value of ISNLD in quantifying the risk of NAFLD‐related adverse outcomes.

### Statistical Analysis

The characteristics of the study population were presented using descriptive statistics. For continuous variables, statistics were reported as mean (standard deviation) or median (interquartile range). Categorical variables were reported as *n* (%). Continuous variables were assessed using the Mann–Whitney *U* test. Categorical variables were evaluated using chi‐square or Fisher's exact tests. ROC curve analysis was performed to assess the predictive efficiency of the ML models. Kaplan–Meier curves for SeLD and other metabolism‐related adverse outcomes were generated based on the ISNLD groups. The association between ISNLD and adverse outcomes was analyzed using Cox proportional hazards regression, with effect sizes reported as HR and accuracy measures (95% CI). Python was used for all modeling analyses. Other analyses were conducted using the R software (version 4.0.2; R Foundation for Statistical Computing, Vienna, Austria). A two‐sided *p* < 0.05 indicated statistical significance for all analyses.

## Conflict of Interest

The authors declare no conflict of interest.

## Author Contributions

L.X., L.Z., J.W., C.H., and Z.Z. contributed equally to this work. L.Z., X.Z., and L.X. performed conceptualization; L.X. and L.L. acquired resources; L.Z. and P.M. performed investigation and methodology; L.Z., P.M., C.W., S.L., and C.H. performed data curation; P.M. and L.Z. performed formal analysis and visualization; C.H., S.L., Y.L., R.L., Q.D., Z.Z., and J.W. performed validation; J.W., L.Z., and Z.Z. wrote the original draft; H.C., L.X., J.W., C.H., and P.M. wrote the original manuscript reviewed, and edited the file; C.H., L.X., X.Z., and L.L. acquired funding; W.L., X.Z., P.M., L.X., and L.L. performed supervision. All authors reviewed the manuscript.

## Supporting information



Supporting Information

## Data Availability

The data that support the findings of this study are available from [UK Biobank]. Restrictions apply to the availability of these data, which were used under a license for this study. Data are available at [https://www.ukbiobank.ac.uk] with the permission of [UK Biobank].
