# Supplementary material for: Development and Validation of Machine Learning‐Based Marker for Early Detection and Prognosis Stratification of Nonalcoholic Fatty Liver Disease
Source: Adv Sci (Weinh). 2025 May 28;12(33):e10527. doi: 10.1002/advs.202410527 (PMC12412496; doi:10.1002/advs.202410527)
Supplement: Supplementary file 1 — Supporting Information [file ADVS-12-e10527-s001.docx]

**Supplementary information**

**Development and Validation of Machine Learning-Based Marker for Early Detection and Prognosis Stratification of Nonalcoholic Fatty Liver Disease**

Lushan Xiao, Lin Zeng, Jiaren Wang, Chang Hong, Ziyong Zhang, Chengkai Wu, Hao Cui, Yan Li, Ruining Li, Shengxing Liang, Qijie Deng, Wenyuan Li, Xuejing Zou, Pengcheng Ma, and Li Liu

Supplementary Methods

Assessment of lifestyle factors 3

References 4

Supplementary Figures

Figure S1. Schematic diagram of risk stratification based on ISNLD. 5

Figure S2. Decision curves analysis and calibration curves for predict NAFLD using the ML model. 6

Figure S3. Top 20 important features for base classifiers computed from feature importance analysis. 7

Figure S4. Comparison of ISNLD between normal (blue) and NAFLD(red). 8

Figure S5. Association of known NAFLD risk factors with ISNLD in the training set.

9

Figure S6. Association of known NAFLD risk factors with ISNLD in the internal test set. 10

Figure S7. Association of known NAFLD risk factors with ISNLD in the external test set. 11

Figure S8. Performance of ISNLD for predicting SeLD in the high-risk group for NAFLD of the internal test set. 12

Figure S9. The cumulative incidences of adverse outcomes among the high-risk group for NAFLD in the internal test set, by ISNLD quartiles. 13

Figure S10. Flowchart of participant selection in two cohorts... 14

Supplementary Tables

Table S1. Characteristics of study participants in the training and internal test sets of UK Biobank. 15

Table S2. The SNPs associated with NAFLD screened out by LASSO. 17

Table S3. Mean performance metrics of single classifiers in the training set across 5-fold cross-validation. 23

Table S4. Comparison of clinical characteristics between low- and high-risk groups for NAFLD in training set. 24

Table S5. Comparison of clinical characteristics between low- and high-risk groups for NAFLD in internal test set. 26

Table S6. Comparison of clinical characteristics between low- and high-risk groups for NAFLD in external test set. 28

Table S7. The associations of ISNLD with metabolism-related outcomes in the high-risk group for NAFLD of training set. 29

Table S8. The associations of ISNLD groups with metabolism-related outcomes in the high-risk group for NAFLD of training set. 30

Table S9. The associations of ISNLD with metabolism-related outcomes in the high-risk group for NAFLD of internal test set. 31

Table S10. The associations of ISNLD groups with metabolism-related outcomes in the high-risk group for NAFLD of internal test set. 32

Table S11. Definition of lifestyle factors in the UK Biobank. 33

Table S12. Coding Algorithms for Defining diseases in the UK Biobank. 34

**Supplementary Methods**

*Assessment of lifestyle factors*

Information on alcohol consumption, smoking status, and physical activity was obtained from the touchscreen questionnaire; diet was derived from the Food Frequency Questionnaire. Alcohol consumption was calculated based on self-reported intake of red wine, white wine, beer, spirits, and fortified wine. Chronic heavy alcohol consumption was defined as 3 or more drinks for women and 4 or more drinks for men on any day (one drink is measured as 8 g ethanol in the U.K.). ^[1]^ Smoking status was dichotomized as smoking vs. non-smoking. Physical activity was measured as minutes per week spent walking or engaged in moderate or vigorous activity according to the International Physical Activity Questionnaire (IPAQ). Regular physical activity was defined as engaging in moderate activity ≥150 min per week, vigorous activity ≥75 min per week, or moderate and vigorous activity ≥150 min/week. ^[2]^ A healthy diet score was generated based on the seven commonly eaten food groups following a more recent definition of ideal intake of dietary components for cardiometabolic health. ^[3]^ A healthy diet was based on intake of at least four of these seven commonly eaten food groups. ^[3]^ Supplementary Table 1 provides additional details regarding the assessment of healthy lifestyle factors.

In the current study, we confirmed four healthy lifestyle factors including no/moderate alcohol consumption, not smoking, regular physical activity, and a healthy diet. Participants were categorized into three groups according to the number of healthy lifestyle factors: (1) unfavorable (0 or 1 healthy lifestyle factors), (2) intermediate (2 factors), and (3) favorable (3 or 4 factors).

*References*

[1] R. Daviet, G. Aydogan, K. Jagannathanet.al., Associations between alcohol consumption and gray and white matter volumes in the UK Biobank, 2022*,* Nat. Commun., *13*, 1175, https://doi.org/10.1038/s41467-022-28735-5.

[2] D. M. Lloyd-Jones, Y. Hong, D. Labartheet.al., Defining and setting national goals for cardiovascular health promotion and disease reduction: the American Heart Association's strategic Impact Goal through 2020 and beyond, 2010*,* Circulation, *121*, 586, https://doi.org/10.1161/CIRCULATIONAHA.109.192703.

[3] D. Mozaffarian, Dietary and Policy Priorities for Cardiovascular Disease, Diabetes, and Obesity: A Comprehensive Review, 2016*,* Circulation, *133*, 187, https://doi.org/10.1161/CIRCULATIONAHA.115.018585.

**Supplementary Figures**

**
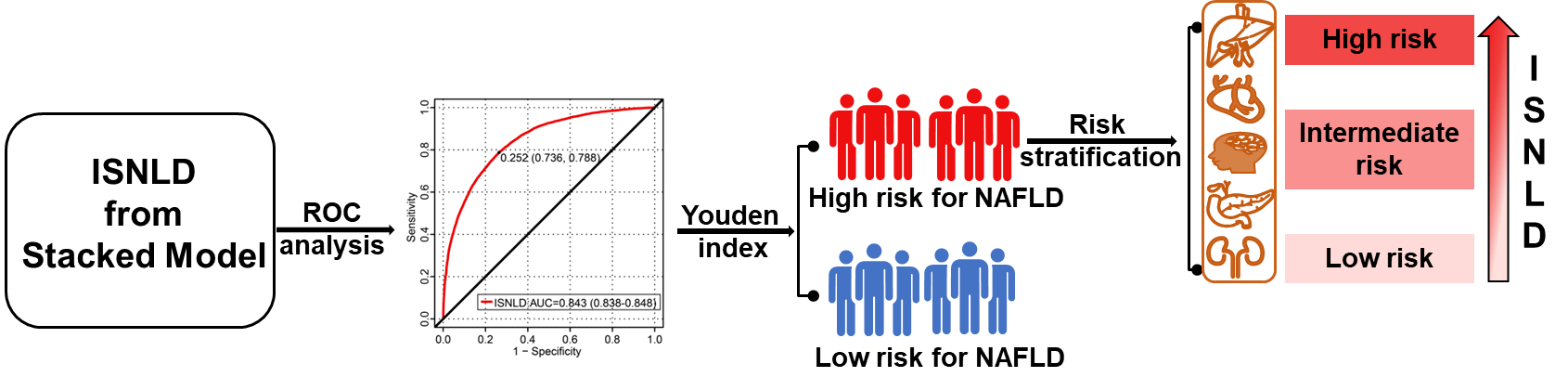
**

**Figure S1.** **Schematic diagram of risk stratification based on ISNLD.**

Abbreviations: ISNLD, in silico score for non-alcoholic fatty liver disease; NAFLD, non-alcoholic fatty liver disease.

**
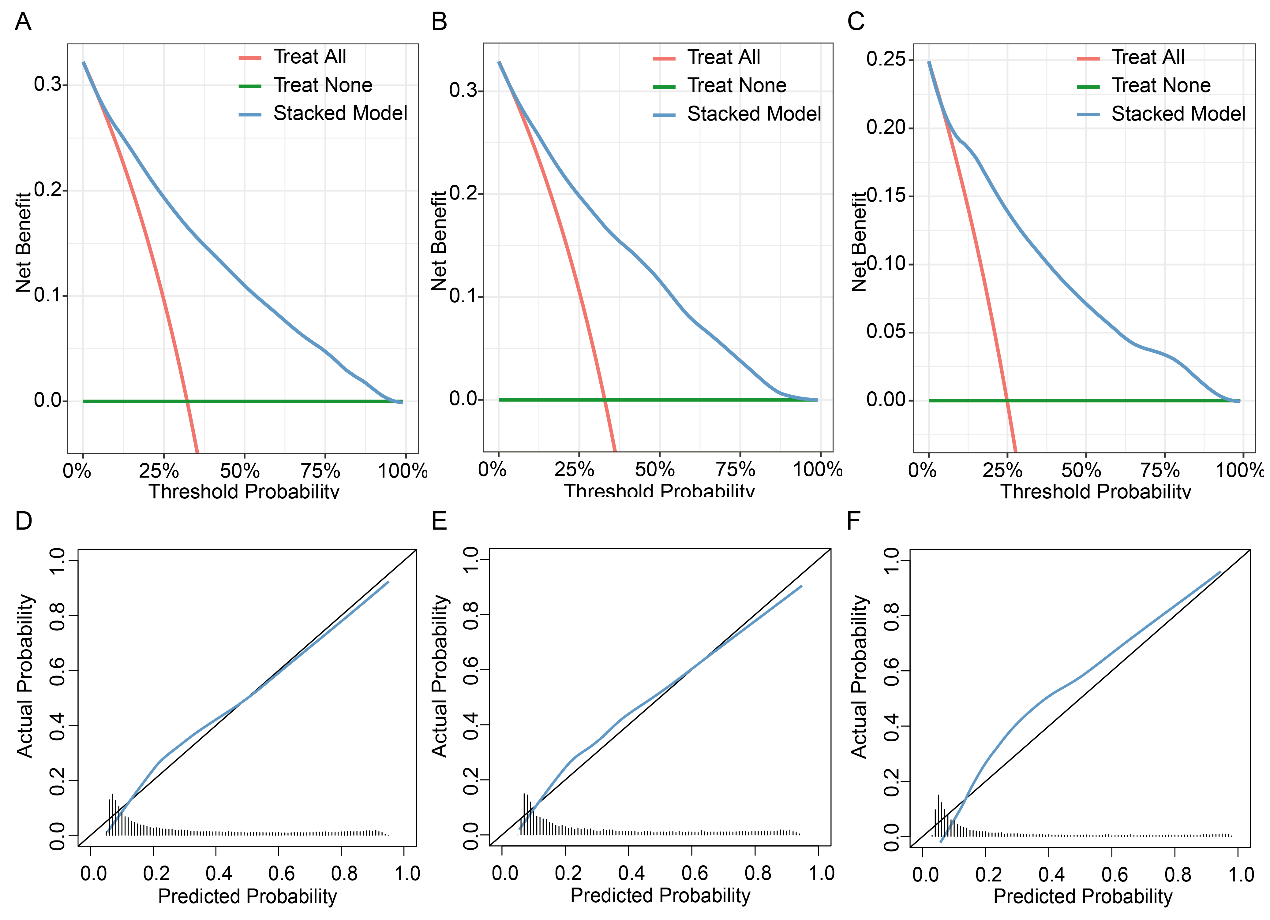
**

**Figure S2.** **Decision curves analysis and calibration curves for predict NAFLD using the ML model.**

(A) Decision curves analysis for predict NAFLD using the ML model in the training set. (B) Decision curves analysis for predict NAFLD using the ML model in the internal test set. (C) Decision curves analysis for predict NAFLD using the ML model in the external test set. (D) Calibration curves for predict NAFLD using the ML model in the training set. (E) Calibration curves for predict NAFLD using the ML model in the internal test set. (F) Calibration curves for predict NAFLD using the ML model in the external test set.


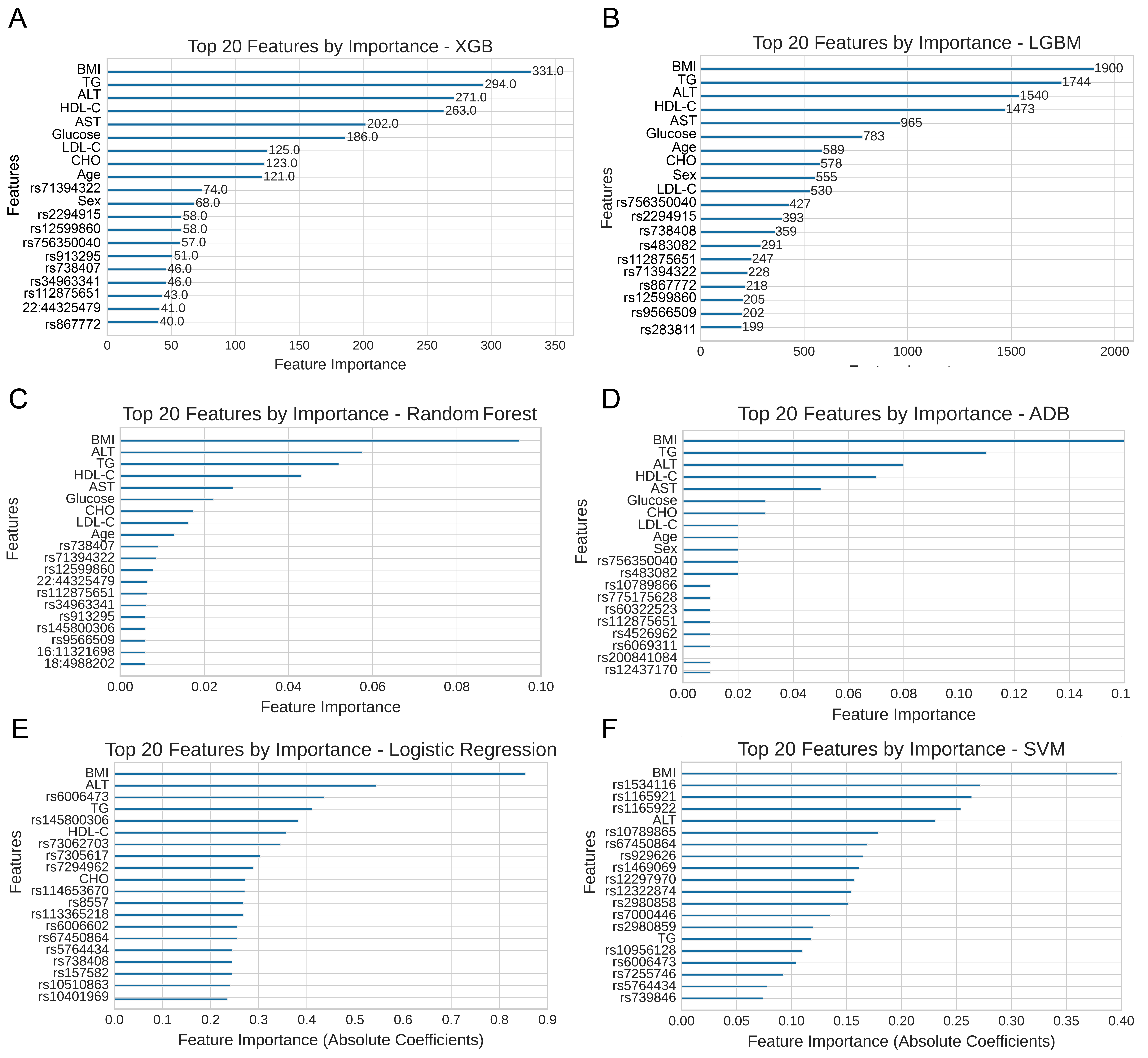


**Figure S3. Top 20 important features for base classifiers computed from feature importance analysis.**

A) XGB, classical extreme gradient boosting. B) LGBM, Light Gradient Boosting Machine. C) Random Forest. D) ADB, Adaptive Boosting. E) Logistic regression. F) SVM, Support Vector Machine.

**
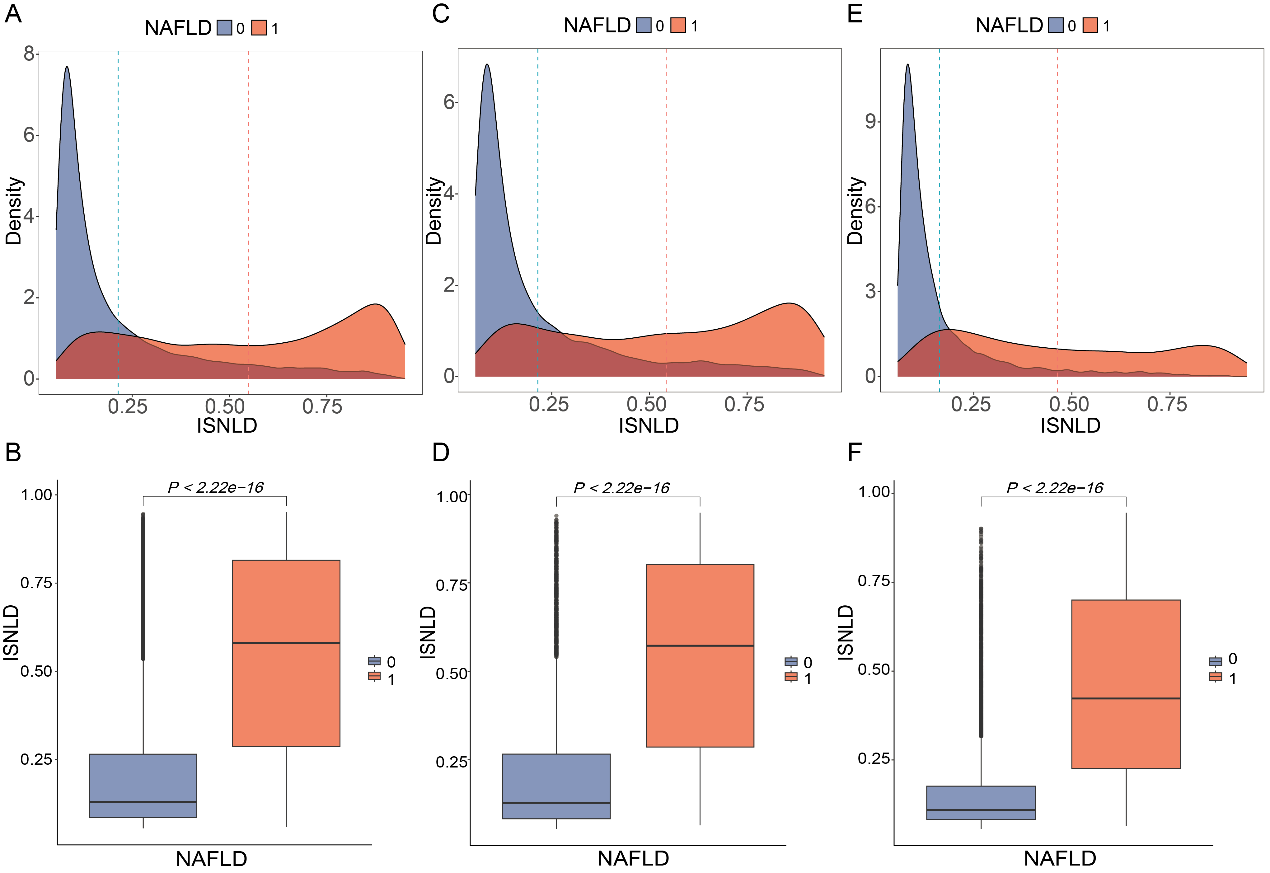
**

**Figure S4. Comparison of ISNLD between normal (blue) and NAFLD(red).**

(A) Density plots of the ISNLD score between normal and NAFLD group in the training set; (B) Comparison of ISNLD percentile between normal and NAFLD group in the training set; (C) Density plots of the ISNLD score between normal and NAFLD group in the internal test set; (D) Comparison of ISNLD percentile between normal and NAFLD group in the internal test set; (E) Density plots of the ISNLD score between normal and NAFLD group in the external set; (F) Comparison of ISNLD percentile between normal and NAFLD group in the external test set.

Abbreviations: ISNLD, in silico score for non-alcoholic fatty liver disease; NAFLD, non-alcoholic fatty liver disease.

**
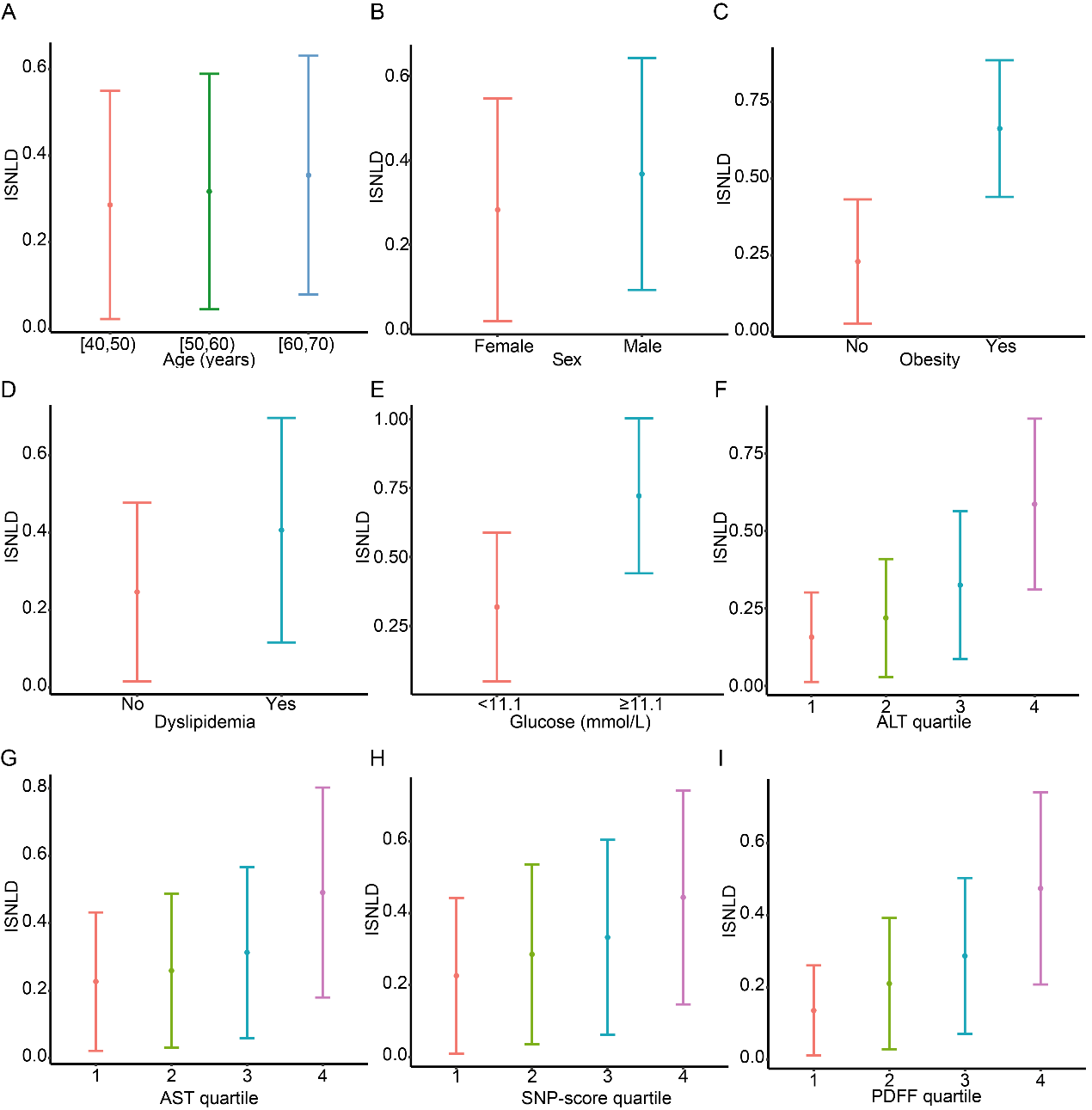
**

**Figure S5.** **Association of known NAFLD risk factors with ISNLD in the training set.**

ISNLD was evaluated for association with known demographic, clinical and genetic risk factors for NAFLD. (A) Age, categorized into three age groups; (B) Sex, categorized into male and female; (C) Obesity, defined as BMI ≥ 30; (D) Dyslipidemia, defined based on LDL-C, CHO, HDL-C, TG levels; (E) Blood glucose; (F) ALT, categorized into quartiles; (G) AST, categorized into quartiles; (H) SNP-score, extracted from the stacked machine learning model for NAFLD based solely on genetic features, categorized into quartiles; (I) PDFF value, categorized into quartiles.

Abbreviations: ISNLD, in silico score for non-alcoholic fatty liver disease; ALT, Alanine Aminotransferase; AST, Aspartate Aminotransferase; SNP, Single nucleotide polymorphisms; PDFF: proton density fat fraction.

**
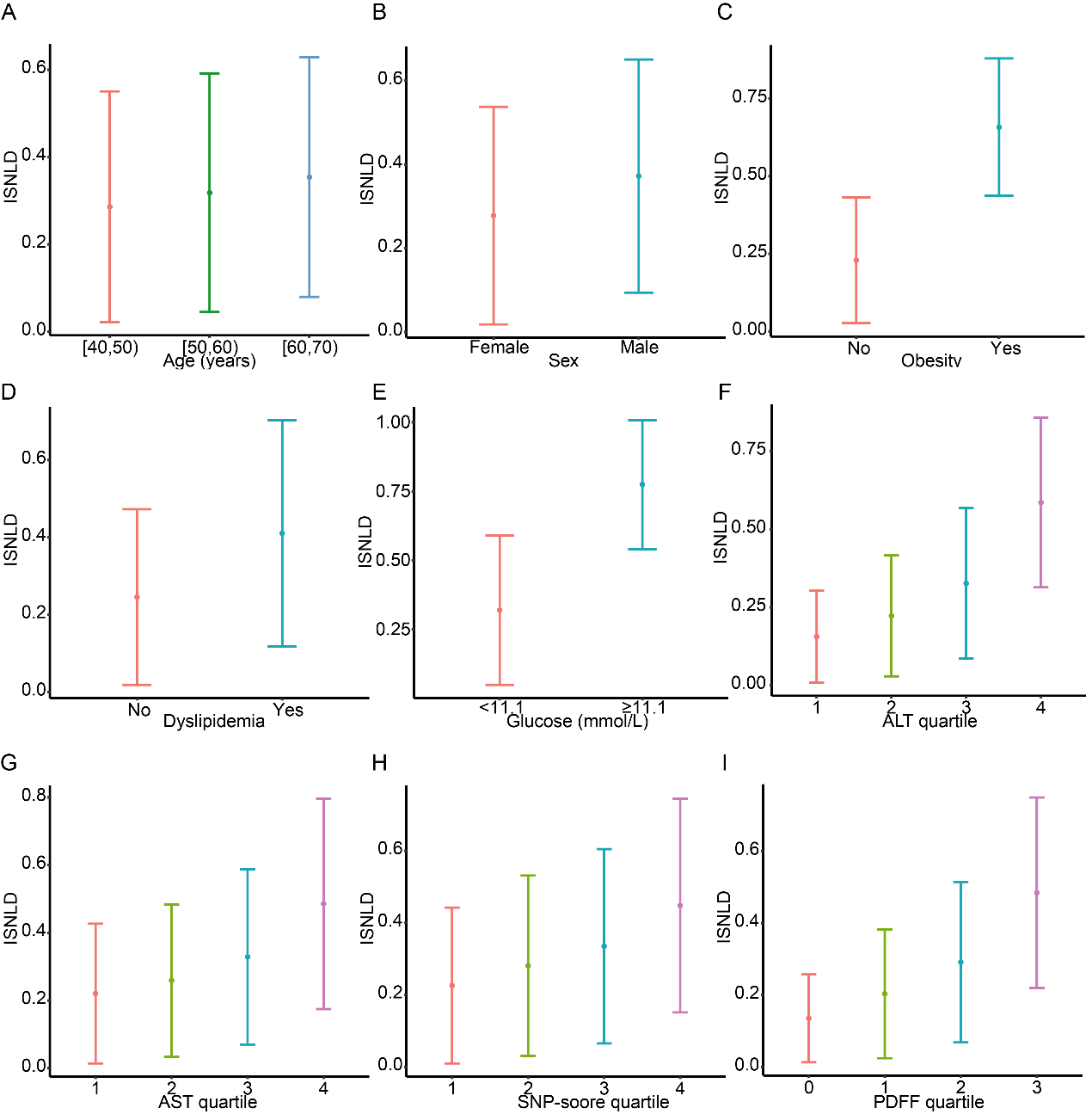
 Figure Figure S6. Association of known NAFLD risk factors with ISNLD in the internal test set.**

ISNLD was evaluated for association with known demographic, clinical and genetic risk factors for NAFLD. (A) Age, categorized into three age groups; (B) Sex, categorized into male and female;(C) Obesity, defined as BMI ≥ 30; (D) Dyslipidemia, defined based on LDL-C, CHO, HDL-C, TG levels; (E) Blood glucose; (F) ALT, categorized into quartiles; (G) AST, categorized into quartiles; (H) SNP-score, extracted from the stacked machine learning model for NAFLD based solely on genetic features, categorized into quartiles; (I) PDFF value, categorized into quartiles.

Abbreviations: ISNLD, in silico score for non-alcoholic fatty liver disease; ALT, Alanine Aminotransferase; AST, Aspartate Aminotransferase; SNP, Single nucleotide polymorphisms; PDFF: proton density fat fraction.

**
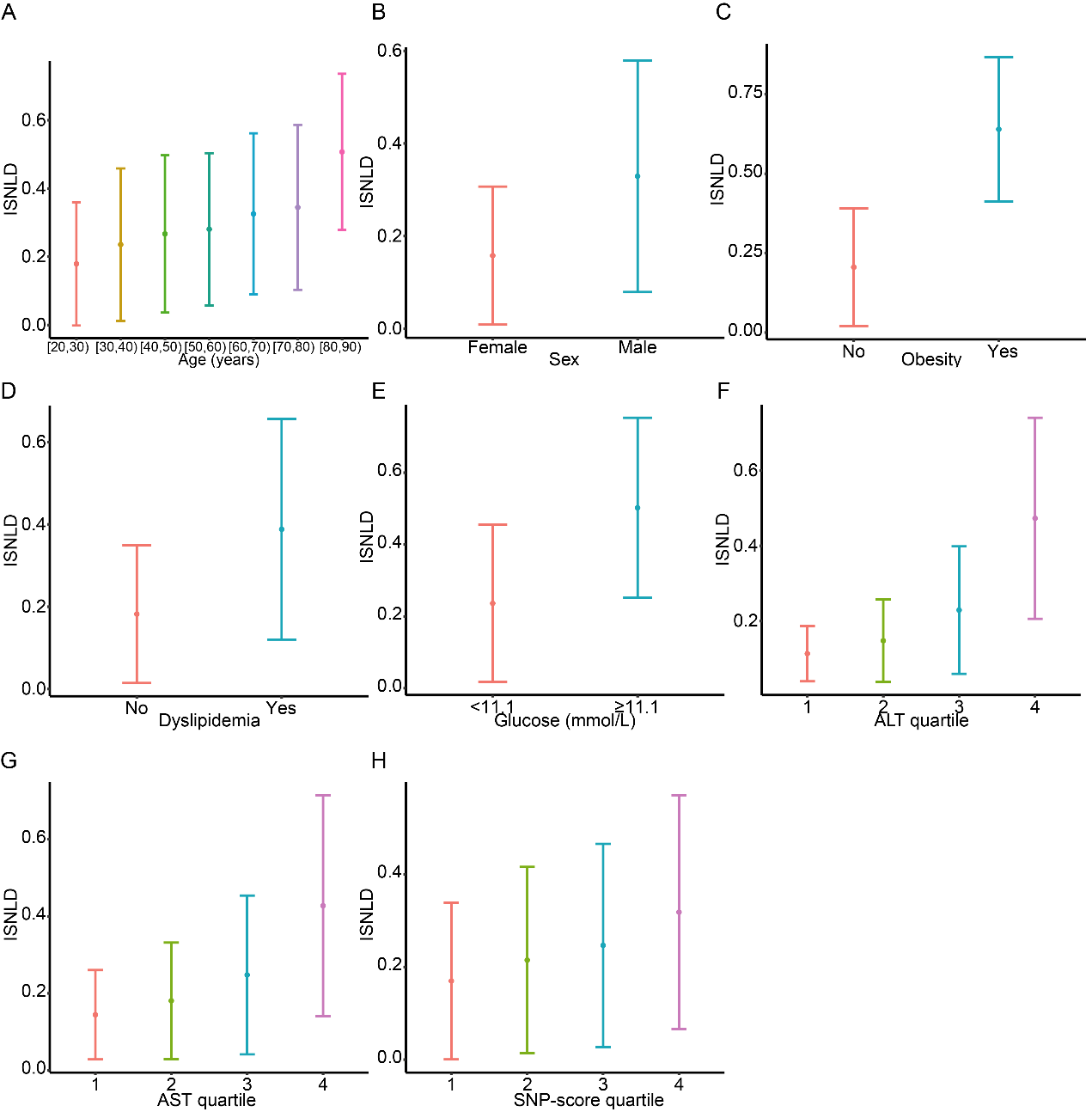
**

**Figure S7. Association of known NAFLD risk factors with ISNLD in the external test set.**

ISNLD was evaluated for association with known demographic, clinical and genetic risk factors for NAFLD. (A) Age, categorized into seven age groups; (B) Sex, categorized into male and female;(C) Obesity, defined as BMI ≥ 30; (D) Dyslipidemia, defined based on LDL-C, CHO, HDL-C, TG levels; (E) Blood glucose; (F) ALT, categorized into quartiles; (G) AST, categorized into quartiles; (H) SNP-score, extracted from the stacked machine learning model for NAFLD based solely on genetic features, categorized into quartiles.

Abbreviations: ISNLD, in silico score for non-alcoholic fatty liver disease; ALT, Alanine Aminotransferase; AST, Aspartate Aminotransferase; SNP, Single nucleotide polymorphisms; PDFF: proton density fat fraction.

**
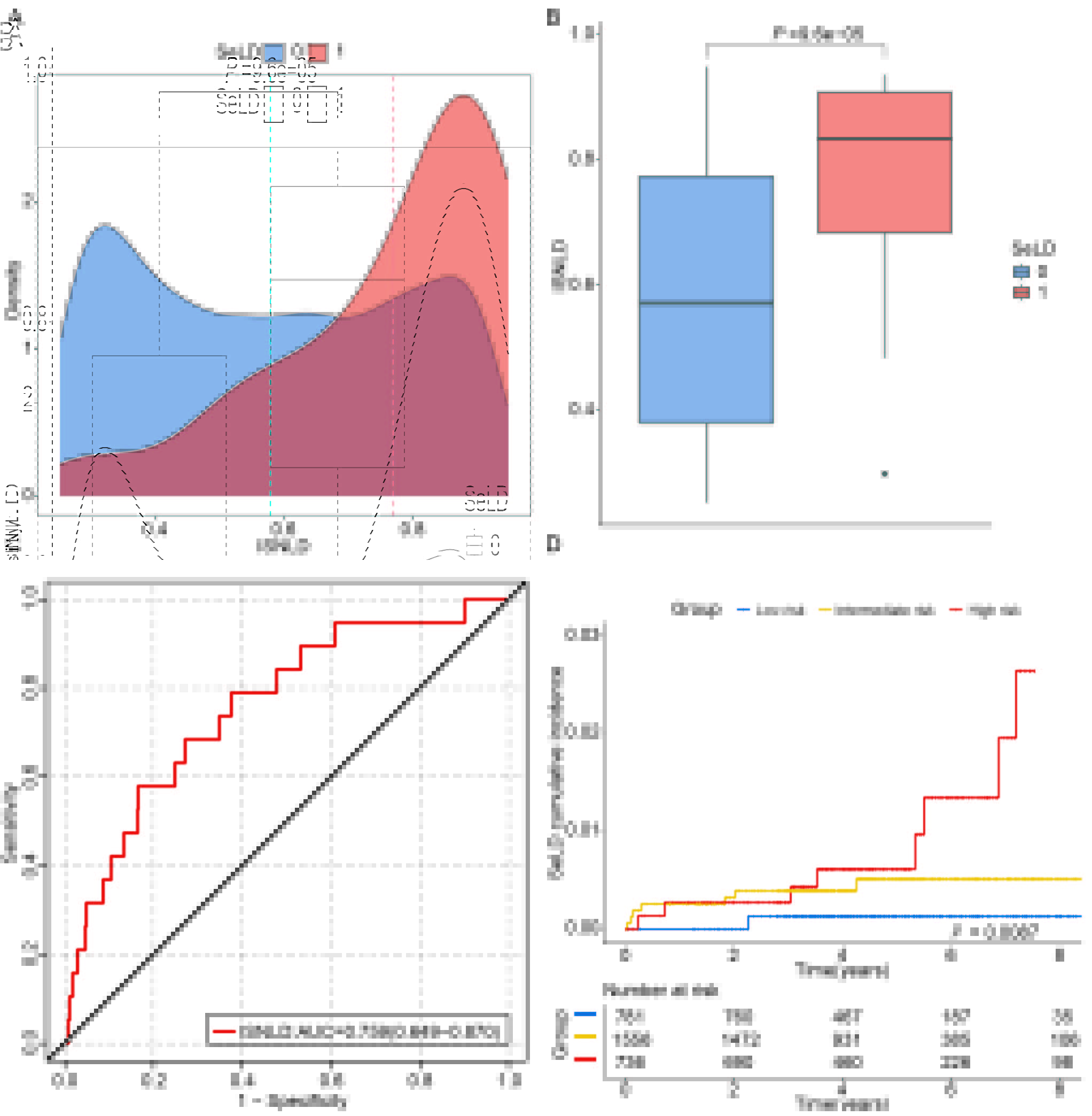
**

**Figure S8. Performance of ISNLD for predicting SeLD in the high-risk group for NAFLD of he internal test set.**

(A) Density plots of the ISNLD score between normal (blue) and SeLD (red) group. (B) Comparison of ISNLD percentile between normal (blue) and SeLD (red) group, P<2.22e-16. (C) The ROC analyses for predicting SeLD with ISNLD. (D) The cumulative risks of developing incident SeLD by ISNLD groups, *P*<0.0001.

Abbreviations: ISNLD, in silico score for non-alcoholic fatty liver disease; SeLD, severe liver disease; AUC, Area Under the Curve; FIB-4, Fibrosis-4 Index.

**
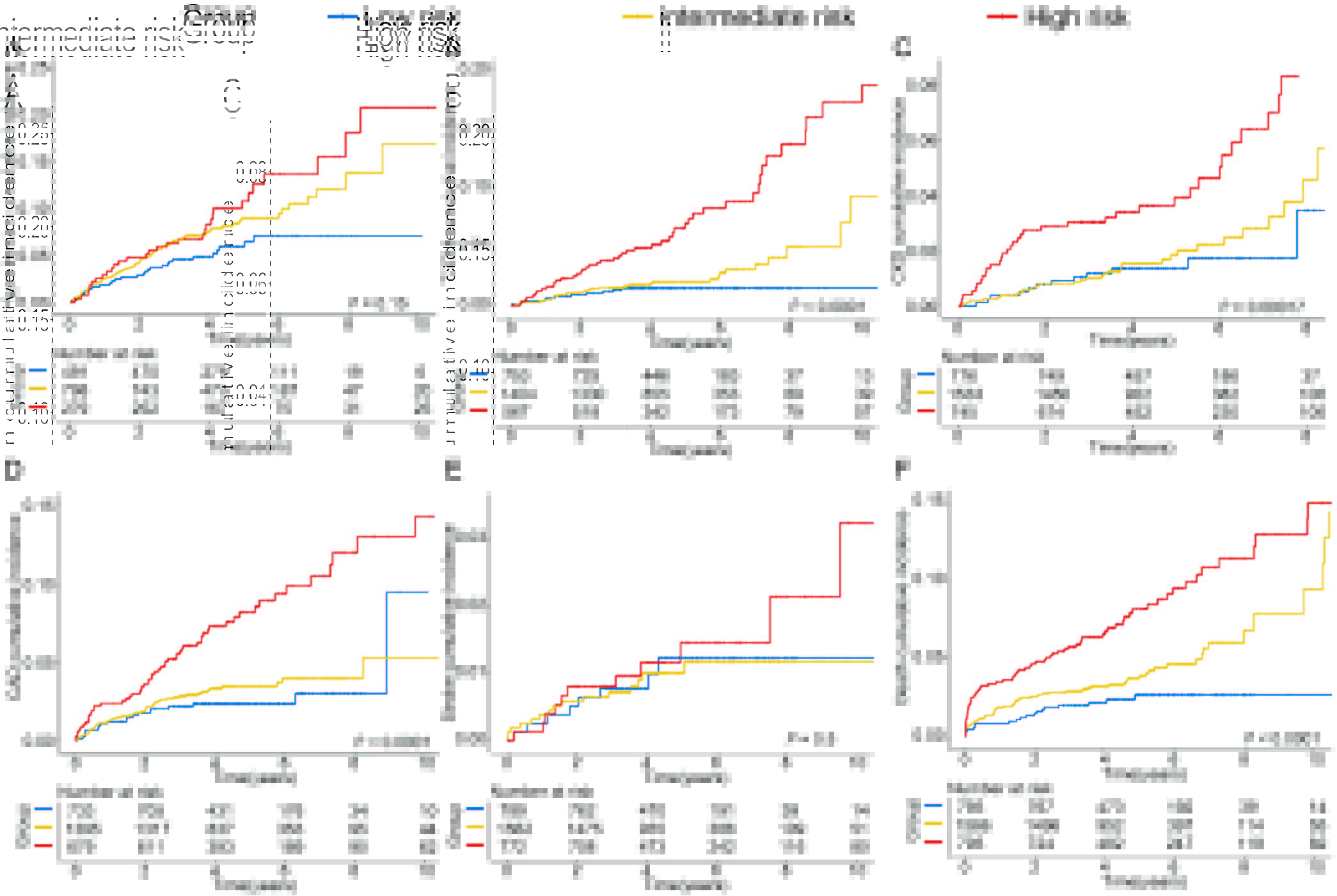
**

**Figure S9. The cumulative incidences of adverse outcomes among the high-risk group for NAFLD in the internal test set, by ISNLD quartiles.**

(A) hypertension; (B) DmT2; (C) CKD; (D) CAD; (E) HF; (F) AF; ISNLD quartile 1 group was set as the reference group.

Abbreviations: ISNLD, in silico score for non-alcoholic fatty liver disease; DmT2, type 2 diabetes; CKD, chronic kidney disease; CAD, coronary artery disease; HF, heart failure; AF, atrial fibrillation/atrial flutter.


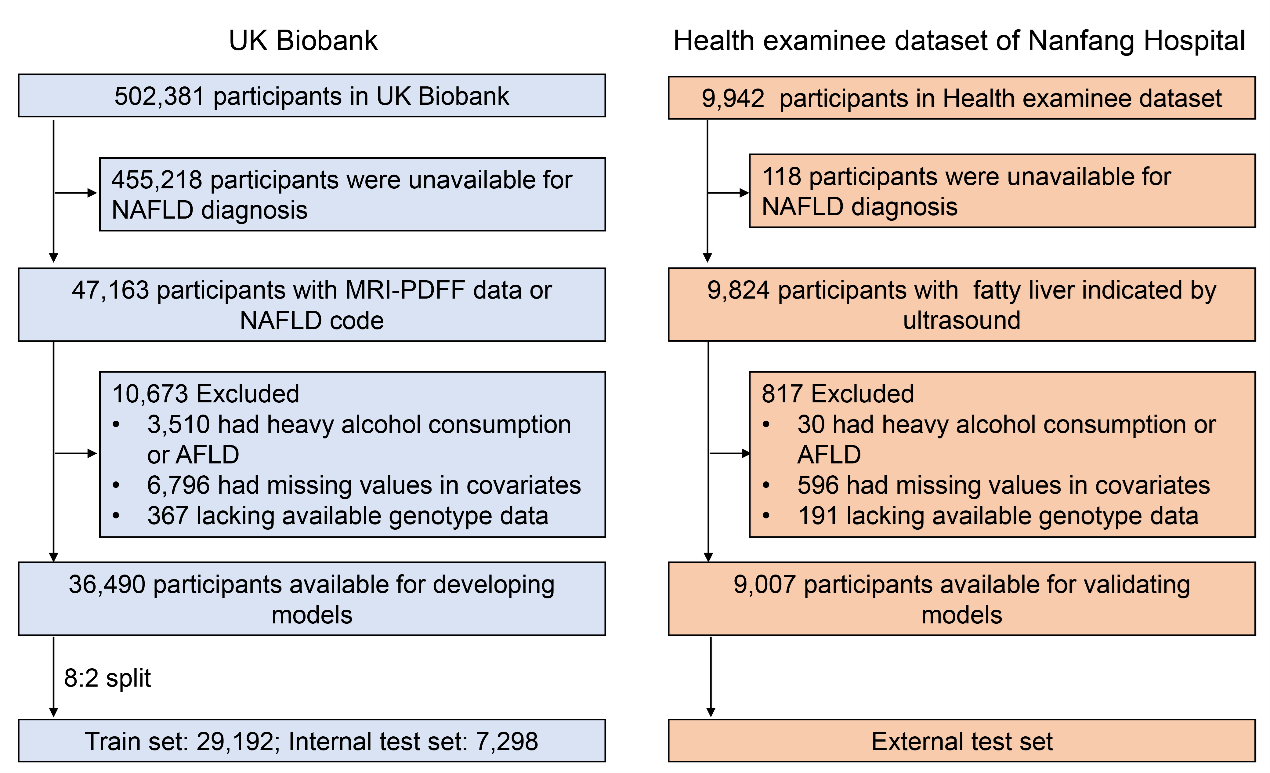


**Figure S10. Flowchart of participant selection in two cohorts.**

**Supplementary Tables**

**Table S1. Characteristics of study participants in the training and internal test sets of UK Biobank.**

| Characteristic | Total (N = 36490) | Train set  ( N= 29192) | Internal test set (N = 7298) | *P*-value |
| --- | --- | --- | --- | --- |
| Age, years | 56 (49, 61) | 56 (49, 61) | 56 (50, 61) | 0.652 |
| Sex, n (%) |  |  |  | 0.111 |
| Female | 19526 (53.51) | 15682 (53.72) | 3844 (52.67) |  |
| Male | 16964 (46.49) | 13510 (46.28) | 3454 (47.33) |  |
| BMI, kg/m^2^ | 26.26 (23.76, 29.36) | 26.26 (23.77, 29.34) | 26.31 (23.73, 29.43) | 0.743 |
| TG, mmol/L | 1.42 (1.00, 2.08) | 1.43 (1.00, 2.08) | 1.41 (1.01, 2.07) | 0.687 |
| LDL-C, mmol/L | 3.51 (2.96, 4.10) | 3.51 (2.96, 4.10) | 3.50 (2.96, 4.09) | 0.471 |
| CHO, mmol/L | 5.63 (4.93, 6.38) | 5.63 (4.93, 6.39) | 5.62 (4.92, 6.37) | 0.517 |
| HDL-C, mmol/L | 1.41 (1.18, 1.68) | 1.41 (1.17, 1.68) | 1.41 (1.18, 1.68) | 0.581 |
| Glu, mmol/L | 4.89 (4.57, 5.25) | 4.89 (4.57, 5.25) | 4.89 (4.57, 5.27) | 0.539 |
| ALT, U/L | 20.01 (15.25, 27.63) | 20.02 (15.26, 27.65) | 19.98 (15.22, 27.54) | 0.412 |
| AST, U/L | 24.30 (20.90, 28.90) | 24.40 (20.90, 28.90) | 24.30 (21.00, 28.90) | 0.748 |
| ISNLD | 0.20 (0.10, 0.51) | 0.20 (0.10, 0.51) | 0.20 (0.10, 0.52) | 0.726 |
| NAFLD | 11803 (32.35) | 9400 (32.20) | 2403 (32.93) | 0.241 |
| Smoking status, n (%) | |  |  | 0.977 |
| Smoker | 10474 (28.70) | 8386 (28.73) | 2088 (28.61) |  |
| Non-smoker | 15240 (41.76) | 12191 (41.76) | 3049 (41.78) |  |
| NA | 10776 (29.53) | 8615 (29.51) | 2161 (29.61) |  |
| Alcohol consumption, n (%) | |  |  | 0.250 |
| Moderate | 16369 (44.86) | 13150 (45.05) | 3219 (44.11) |  |
| Never/mild | 9345 (25.61) | 7427 (25.44) | 1918 (26.28) |  |
| NA | 10776 (29.53) | 8615 (29.51) | 2161 (29.61) |  |
| Physical activity, n (%) | |  |  | 0.957 |
| Inactive | 11739 (32.17) | 9386 (32.15) | 2353 (32.24) |  |
| Active | 13975 (38.30) | 11191 (38.34) | 2784 (38.15) |  |
| NA | 10776 (29.53) | 8615 (29.51) | 2161 (29.61) |  |
| Diet, n (%) ^a^ |  |  |  | 0.809 |
| Unhealthy | 9888 (27.10) | 7893 (27.04) | 1995 (27.34) |  |
| Healthy | 15826 (43.37) | 12684 (43.45) | 3142 (43.05) |  |
| NA | 10776 (29.53) | 8615 (29.51) | 2161 (29.61) |  |
| Lifestyle, n (%) ^b^ |  |  |  | 0.955 |
| Unfavorable | 7057 (19.34) | 5656 (19.38) | 1401 (19.20) |  |
| Intermediate | 9370 (25.68) | 7506 (25.71) | 1864 (25.54) |  |
| Favorable | 9287 (25.45) | 7415 (25.40) | 1872 (25.65) |  |
| NA | 10776 (29.53) | 8615 (29.51) | 2161 (29.61) |  |
| SeLD, n (%) |  |  |  | 0.485 |
| No | 35793 (98.09) | 28637 (98.10) | 7156 (98.05) |  |
| Yes | 116 (0.32) | 97 (0.33) | 19 (0.26) |  |
| NA | 581 (1.59) | 458 (1.57) | 123 (1.69) |  |
| Hypertension, n (%) | |  |  | 0.856 |
| No | 22224 (60.90) | 17795 (60.96) | 4429 (60.69) |  |
| Yes | 1107 (3.03) | 889 (3.05) | 218 (2.99) |  |
| NA | 13159 (36.06) | 10508 (36.00) | 2651 (36.33) |  |
| DmT2, n (%) |  |  |  | 0.461 |
| No | 33416 (91.58) | 26740 (91.60) | 6676 (91.48) |  |
| Yes | 543 (1.49) | 423 (1.45) | 120 (1.64) |  |
| NA | 2531 (6.94) | 2029 (6.95) | 502 (6.88) |  |
| CKD, n (%) |  |  |  | 0.928 |
| No | 35071 (96.11) | 28057 (96.11) | 7014 (96.11) |  |
| Yes | 654 (1.79) | 526 (1.80) | 128 (1.75) |  |
| NA | 765 (2.10) | 609 (2.09) | 156 (2.14) |  |
| CAD, n (%) |  |  |  | 0.226 |
| No | 33010 (90.46) | 26428 (90.53) | 6582 (90.19) |  |
| Yes | 879 (2.41) | 683 (2.34) | 196 (2.69) |  |
| NA | 2601 (7.13) | 2081 (7.13) | 520 (7.13) |  |
| HF, n (%) |  |  |  | 0.262 |
| No | 35429 (97.09) | 28341 (97.08) | 7088 (97.12) |  |
| Yes | 447 (1.22) | 369 (1.26) | 78 (1.07) |  |
| NA | 614 (1.68) | 482 (1.65) | 132 (1.81) |  |
| AF, n (%) |  |  |  | 0.690 |
| No | 34378 (94.21) | 27516 (94.26) | 6862 (94.03) |  |
| Yes | 768 (2.10) | 613 (2.10) | 155 (2.12) |  |
| NA | 1344 (3.68) | 1063 (3.64) | 281 (3.85) |  |
| All-cause death, n (%) | |  |  | 0.815 |
| No | 35358 (96.90) | 28290 (96.91) | 7068 (96.85) |  |
| Yes | 1132 (3.10) | 902 (3.09) | 230 (3.15) |  |

^a^ Diet pattern included seven dietary components: fruits, vegetables, whole grains, refined grains, fish, unprocessed meat, and processed meat;

^b^ Lifestyle index was created by four healthy lifestyle factors: never/moderate alcohol consumption, no smoking, regular physical activity, and a healthy diet. Participants were categorized into three groups according to the number of healthy lifestyle factors: (1) unfavorable (0 or 1), (2) intermediate (any 2), and (3) favorable (3 or 4); Abbreviations: NAFLD, nonalcoholic fatty liver disease; BMI, Body mass index; TG, Triglyceride; CHO, Cholesterol; LDL-C, Low-density lipoprotein-cholesterol; HDL-C, High-density lipoprotein-cholesterol; Glu, blood glucose; ALT, alanine aminotransferase; AST, aspartate aminotransferase; ISNLD, in-silico score for NAFLD; AF, Atrial fibrillation/Atrial flutter; CAD, coronary artery disease; HF, heart failure; DmT2, Type 2 diabetes; CKD, chronic kidney disease;

**Table S2.** **The SNPs associated with NAFLD screened out by LASSO.**

| Chrom | Rsid | Linear closest gene | REF | ALT | OR | *P-*value |
| --- | --- | --- | --- | --- | --- | --- |
| 1 | rs2642438 | MTARC1 | A | G | 0.859 | 1.840E-14 |
| 1 | rs116022902 |  | G | A | 1.253 | 2.560E-03 |
| 1 | rs12077210 | LEPR/ LEPROT | C | T | 1.124 | 7.825E-03 |
| 1 | rs61830266 | MTARC1 | A | G | 1.059 | 1.573E-03 |
| 1 | rs10779416 | MTARC1 | G | C | 1.040 | 3.373E-02 |
| 1 | rs2642442 | MTARC1 | C | T | 0.871 | 8.657E-13 |
| 1 | rs867772 | MTARC1 | A | G | 0.868 | 3.724E-13 |
| 1 | rs148429571 | HLX-AS1 | A | G | 1.147 | 4.046E-02 |
| 1 | rs17005517 | LOC101929713 | C | A | 1.035 | 8.345E-02 |
| 1 | rs34963341 | CELA3B | C | A | 0.946 | 6.924E-03 |
| 2 | rs1919127 | C2orf16 | T | C | 1.105 | 6.983E-07 |
| 2 | rs2068834 | ZNF512 | T | C | 1.098 | 2.409E-06 |
| 2 | rs1260326 | GCKR | T | C | 1.115 | 1.750E-09 |
| 2 | rs67802835 | PAX8 | A | G | 1.052 | 3.776E-02 |
| 2 | rs113180498 | COMMD1 | G | A | 0.944 | 2.422E-01 |
| 2 | rs17671252 | LOC105373757 | C | T | 1.028 | 1.812E-01 |
| 2 | rs778380010 |  | AAGG | A | 0.924 | 2.092E-05 |
| 2 | rs767012 |  | C | T | 1.037 | 1.174E-01 |
| 2 | rs1035102 |  | G | T | 1.056 | 2.415E-03 |
| 2 | 2:227036698_AT_A | LOC646736 | AT | A | 0.965 | 4.434E-02 |
| 2 | 2:227111435_TTA_T | | TTA | T | 0.918 | 4.205E-06 |
| 3 | rs140216924 | KIF9-AS1 | C | G | 0.957 | 6.164E-02 |
| 3 | rs6442060 | SETD2 | A | T | 0.957 | 6.206E-02 |
| 3 | rs114653670 |  | G | A | 1.107 | 9.949E-03 |
| 3 | rs1165921 |  | A | T | 1.024 | 2.111E-01 |
| 3 | rs732289 |  | A | G | 1.070 | 3.839E-04 |
| 3 | rs10510863 | LOC105377114 | G | A | 1.102 | 1.375E-02 |
| 3 | rs62236834 |  | A | C | 1.048 | 3.001E-01 |
| 3 | rs1165922 |  | G | A | 1.024 | 1.998E-01 |
| 4 | rs9992651 | HSD17B13 | G | A | 0.974 | 1.961E-01 |
| 4 | rs13118664 | HSD17B13 | A | T | 0.974 | 1.953E-01 |
| 4 | rs138262191 |  | T | C | 1.275 | 1.481E-02 |
| 4 | rs77449637 |  | C | G | 1.074 | 5.185E-03 |
| 4 | rs6847508 |  | T | G | 1.021 | 2.491E-01 |
| 4 | rs6856110 |  | C | G | 1.021 | 2.406E-01 |
| 4 | rs2389918 |  | A | T | 1.021 | 2.544E-01 |
| 4 | rs7694855 |  | G | A | 1.075 | 4.956E-03 |
| 5 | rs34417 | FBXL17 | G | C | 1.027 | 1.783E-01 |
| 5 | rs112758421 |  | C | A | 1.039 | 5.568E-02 |
| 5 | rs929626 | EBF1 | A | G | 1.025 | 1.661E-01 |
| 5 | rs1469069 | EBF1 | T | G | 1.025 | 1.587E-01 |
| 5 | rs766818705 |  | ATTCT | A | 1.051 | 1.183E-02 |
| 5 | rs11953893 |  | T | C | 1.050 | 1.247E-02 |
| 6 | rs78912080 | LRRC16A | A | G | 1.144 | 7.921E-05 |
| 6 | rs28393611 | LRRC16A | C | T | 1.072 | 3.234E-04 |
| 6 | rs4512236 | LOC101928923 | G | T | 0.973 | 1.199E-01 |
| 6 | rs572494 | GCLC | T | C | 1.040 | 2.715E-02 |
| 6 | rs9384407 | LOC101928923 | T | C | 0.977 | 1.929E-01 |
| 6 | rs6936723 | LOC101928923 | C | T | 0.976 | 1.862E-01 |
| 6 | rs9383788 | LOC101928923 | C | T | 0.972 | 1.192E-01 |
| 7 | rs111832199 |  | C | T | 1.139 | 1.918E-04 |
| 7 | rs79004373 | IMMP2L | T | C | 1.187 | 7.475E-04 |
| 8 | rs13252776 | WDYHV1 | C | T | 0.965 | 5.751E-02 |
| 8 | rs112875651 |  | G | A | 0.862 | 1.283E-15 |
| 8 | rs2980888 |  | T | C | 1.119 | 5.233E-09 |
| 8 | rs10956128 | WDYHV1 | C | T | 0.965 | 5.977E-02 |
| 8 | rs13252934 | WDYHV1 | G | A | 0.965 | 5.775E-02 |
| 8 | rs2980858 |  | T | C | 1.112 | 3.121E-08 |
| 8 | rs2980870 |  | A | G | 1.106 | 4.735E-08 |
| 8 | rs2954028 |  | T | C | 1.100 | 5.982E-07 |
| 8 | rs2954031 |  | G | T | 0.884 | 6.096E-12 |
| 8 | rs4871603 |  | C | T | 1.114 | 6.005E-09 |
| 8 | rs2954027 |  | T | A | 0.888 | 2.980E-11 |
| 8 | rs7000446 | WDYHV1 | C | A | 0.965 | 5.825E-02 |
| 8 | rs2980859 |  | C | G | 1.111 | 4.154E-08 |
| 8 | rs2954025 |  | C | T | 1.106 | 5.188E-08 |
| 8 | rs10808546 |  | C | T | 0.875 | 9.874E-14 |
| 8 | rs60322523 |  | T | C | 1.127 | 1.702E-02 |
| 8 | rs13253961 | WDYHV1 | A | G | 0.965 | 5.683E-02 |
| 8 | rs13263438 | WDYHV1 | G | A | 0.963 | 4.346E-02 |
| 8 | 8:126483843_TAA_T | | TAA | T | 1.103 | 3.245E-07 |
| 9 | rs10733608 | AKNA | G | T | 0.927 | 2.272E-05 |
| 9 | rs7043196 | AKNA | C | T | 0.932 | 6.848E-05 |
| 9 | rs2763188 | AKNA | C | T | 1.059 | 3.742E-03 |
| 9 | rs4877739 | FRMD3-AS1 | C | T | 0.944 | 3.104E-03 |
| 10 | rs7916514 |  | A | G | 0.941 | 1.928E-03 |
| 10 | rs11195857 |  | G | A | 0.933 | 2.477E-03 |
| 10 | rs11259258 | FAM107B | A | C | 0.967 | 1.297E-01 |
| 10 | rs55875049 |  | A | G | 0.934 | 2.698E-03 |
| 10 | rs11015882 | LINC02680 | T | C | 1.061 | 8.829E-02 |
| 10 | rs913295 | FAM107B | G | T | 0.995 | 8.061E-01 |
| 10 | rs7903554 | GRID1-AS1 | C | G | 1.073 | 5.759E-02 |
| 11 | rs733454 | LOC124902719 | C | T | 1.061 | 4.480E-02 |
| 11 | rs10789865 | LOC283140 | C | T | 1.071 | 3.059E-03 |
| 11 | rs4937254 | LOC283140 | C | G | 1.069 | 4.207E-03 |
| 11 | rs1534116 | LOC283140 | A | T | 1.071 | 3.175E-03 |
| 11 | rs373143154 | ARHGAP32 | T | TCAC | 1.040 | 6.758E-02 |
| 11 | rs200841084 |  | G | GAC | 1.512 | 4.511E-03 |
| 11 | rs10789866 | LOC283140 | C | T | 1.071 | 3.171E-03 |
| 12 | rs7305617 | GTSF1 | G | C | 1.075 | 1.544E-02 |
| 12 | rs55829819 | GNPTAB | T | G | 1.061 | 2.763E-02 |
| 12 | rs7294962 |  | T | C | 1.087 | 1.487E-03 |
| 12 | rs1024587 | GNPTAB | T | C | 1.061 | 2.697E-02 |
| 12 | rs12322874 | LOC102724050 | A | G | 1.072 | 1.914E-02 |
| 12 | rs12317666 | LOC102724050 | G | A | 1.070 | 2.421E-02 |
| 12 | rs73156618 | ANO4 | T | C | 1.073 | 1.306E-02 |
| 12 | rs541983520 |  | A | AT | 1.075 | 7.426E-03 |
| 12 | rs12297970 | LOC102724050 | T | C | 1.072 | 2.001E-02 |
| 12 | rs139133056 | GTSF1 | G | GT | 1.076 | 1.471E-02 |
| 12 | rs145800306 | LINC02617 | G | GA | 0.949 | 1.347E-02 |
| 12 | rs377026675 | GNPTAB | GAATCATTGTTTTTTAA | G | 1.058 | 3.658E-02 |
| 12 | rs12306350 | GTSF1 | T | C | 1.072 | 1.876E-02 |
| 12 | rs12312643 | GTSF1 | A | G | 1.072 | 1.940E-02 |
| 12 | rs4764656 |  | G | A | 1.088 | 1.319E-03 |
| 12 | rs547223695 |  | C | CT | 1.086 | 1.511E-03 |
| 12 | rs73062703 |  | A | G | 0.952 | 1.917E-02 |
| 12 | rs4981025 | STAB2 | A | G | 0.976 | 2.142E-01 |
| 12 | 12:102162520_ACTC_A | GNPTAB | ACTC | A | 1.061 | 2.852E-02 |
| 12 | 12:102243931_TA_T | | TA | T | 1.088 | 1.295E-03 |
| 13 | rs9566509 |  | A | G | 1.061 | 1.230E-03 |
| 13 | rs17088550 |  | A | G | 1.089 | 8.762E-02 |
| 14 | rs12437170 | LOC107984696 | C | T | 1.033 | 1.580E-01 |
| 14 | rs112277418 |  | G | A | 0.991 | 8.016E-01 |
| 14 | rs4900963 |  | T | G | 1.026 | 3.323E-01 |
| 14 | rs111531727 |  | C | CTTTTT | 1.023 | 4.060E-01 |
| 14 | rs4900419 |  | G | A | 1.052 | 6.070E-03 |
| 14 | rs17548965 | LOC107984696 | A | G | 1.034 | 1.424E-01 |
| 14 | rs11851456 |  | G | T | 1.030 | 1.331E-01 |
| 14 | rs61981294 |  | C | A | 1.033 | 7.241E-02 |
| 14 | rs6575699 |  | C | T | 1.047 | 1.088E-02 |
| 14 | rs17555271 | NPAS3 | A | G | 0.859 | 4.212E-04 |
| 14 | rs4509936 |  | G | A | 1.046 | 2.128E-02 |
| 14 | rs4526962 |  | T | C | 1.050 | 6.772E-03 |
| 15 | rs1871362 | FMN1 | C | T | 0.992 | 6.487E-01 |
| 15 | rs12898728 |  | C | T | 0.949 | 1.021E-02 |
| 15 | rs8557 | MYO9A | T | C | 0.939 | 1.807E-03 |
| 15 | 15:60898653_GT_G | RORA/LOC101928784 | GT | G | 1.058 | 1.480E-02 |
| 16 | rs12599860 | GSE1 | T | C | 0.969 | 9.102E-02 |
| 16 | rs12597513 |  | G | A | 1.027 | 4.194E-01 |
| 16 | rs12917993 |  | C | G | 1.023 | 3.587E-01 |
| 16 | rs71394322 | RBFOX1 | G | GAATAAATAAATAAATAAATA | 1.014 | 4.625E-01 |
| 16 | rs8061221 | PDXDC2P | G | A | 0.967 | 9.978E-02 |
| 16 | 16:11321698_GT_G | | GT | G | 1.016 | 3.681E-01 |
| 17 | rs9903403 |  | G | T | 0.990 | 6.296E-01 |
| 17 | rs1396515 |  | G | C | 1.027 | 1.256E-01 |
| 17 | rs1396514 |  | C | T | 1.028 | 1.247E-01 |
| 17 | rs766752 |  | G | A | 1.026 | 1.482E-01 |
| 17 | rs9890689 |  | G | C | 1.026 | 1.481E-01 |
| 18 | rs7228235 |  | C | T | 0.994 | 7.349E-01 |
| 18 | 18:4988202_TC_T | | TC | T | 0.956 | 2.198E-02 |
| 19 | rs71352238 | TOMM40 | T | C | 0.773 | 8.832E-23 |
| 19 | rs56255430 | LOC124904656 | A | C | 1.629 | 1.508E-56 |
| 19 | rs7252888 | NDUFA13/ TSSK6 | G | A | 1.266 | 8.098E-26 |
| 19 | rs157581 | TOMM40 | T | C | 0.806 | 1.832E-22 |
| 19 | rs7255746 | ELOF1/ LOC101928434 | A | G | 1.073 | 5.113E-03 |
| 19 | rs11668386 | GATAD2A | A | G | 1.372 | 4.381E-34 |
| 19 | rs483082 | APOC1 | G | T | 0.815 | 2.194E-21 |
| 19 | rs769449 | APOE | G | A | 0.746 | 7.484E-25 |
| 19 | rs157582 | TOMM40 | C | T | 0.801 | 1.427E-23 |
| 19 | rs61061000 | MEF2BNB-MEF2B | C | T | 1.231 | 3.487E-14 |
| 19 | rs12972156 | PVRL2 | C | G | 0.782 | 1.136E-20 |
| 19 | rs12972970 | PVRL2 | G | A | 0.782 | 1.208E-20 |
| 19 | rs283815 | PVRL2 | A | G | 0.809 | 9.092E-22 |
| 19 | rs283811 | PVRL2 | A | G | 0.808 | 1.158E-21 |
| 19 | rs6857 | PVRL2 | C | T | 0.780 | 8.998E-24 |
| 19 | rs73002960 | GATAD2A | C | T | 1.391 | 1.326E-35 |
| 19 | rs775175628 | GATAD2A | TATCTTATATTAA | T | 1.255 | 2.879E-23 |
| 19 | rs73004966 | PBX4 | C | T | 1.594 | 1.150E-52 |
| 19 | rs8110999 | CNN1 | C | A | 1.073 | 4.871E-03 |
| 19 | rs58542926 | TM6SF2 | C | T | 1.687 | 3.130E-59 |
| 19 | rs4808196 | LOC124904656 | G | A | 1.268 | 4.713E-26 |
| 19 | rs2304128 | GMIP | G | T | 1.582 | 8.359E-48 |
| 19 | rs8105984 | HAPLN4/ TM6SF2 | T | C | 1.271 | 2.740E-25 |
| 19 | rs56131196 | APOC1 | G | A | 0.811 | 5.196E-19 |
| 19 | rs8105094 | HAPLN4/ TM6SF2 | C | T | 1.271 | 2.753E-25 |
| 19 | rs8107974 | SUGP1 | A | T | 1.680 | 6.824E-60 |
| 19 | rs150824230 | PBX4 | G | A | 1.602 | 3.666E-52 |
| 19 | rs756350040 | HAPLN4 | TGACA | T | 1.704 | 5.109E-59 |
| 19 | rs12979148 | SUGP1 | T | C | 1.328 | 9.130E-29 |
| 19 | rs73004975 | PBX4 | A | G | 1.592 | 1.639E-52 |
| 19 | rs2240117 | SUGP1 | C | T | 1.328 | 1.037E-28 |
| 19 | rs12608729 | PBX4 | C | T | 1.594 | 1.683E-52 |
| 19 | rs10401969 | SUGP1 | T | C | 1.672 | 6.047E-59 |
| 19 | rs143988316 |  | C | T | 1.595 | 1.509E-51 |
| 19 | rs113365218 | GATAD2A | G | A | 1.371 | 7.029E-32 |
| 19 | rs117737135 | ZNF100 | A | C | 1.073 | 1.626E-01 |
| 19 | rs739846 | SUGP1 | G | A | 1.671 | 2.024E-58 |
| 19 | rs2285626 | MAU2 | C | T | 1.397 | 2.008E-38 |
| 19 | rs17216588 |  | C | T | 1.600 | 1.123E-51 |
| 19 | rs56273306 | GATAD2A | T | C | 1.391 | 1.088E-35 |
| 19 | rs751858 | GATAD2A | G | C | 1.269 | 1.313E-26 |
| 19 | rs56408111 | ZNF101 | T | C | 1.447 | 4.852E-33 |
| 19 | rs2285628 | MAU2 | T | A | 1.247 | 2.046E-22 |
| 19 | rs73004951 | PBX4 | C | T | 1.596 | 2.450E-52 |
| 19 | 19:11675424_CA_C | | CA | C | 1.074 | 4.847E-03 |
| 19 | 19:19438110_GTATT_G | MAU2 | GTATT | G | 1.190 | 4.215E-17 |
| 19 | 19:19450254_CA_C | MAU2 | CA | C | 1.383 | 1.251E-33 |
| 19 | 19:19756073_AGCC_A | ATP13A1 | AGCC | A | 1.484 | 1.842E-31 |
| 20 | rs17302162 |  | T | G | 1.055 | 2.340E-02 |
| 20 | rs111802259 |  | A | AT | 0.948 | 3.217E-03 |
| 20 | rs17302134 |  | T | C | 1.055 | 2.372E-02 |
| 20 | rs6142067 |  | C | T | 0.947 | 2.518E-03 |
| 20 | rs6069315 |  | T | C | 1.056 | 2.174E-02 |
| 20 | rs6069311 |  | C | A | 1.054 | 2.573E-02 |
| 22 | rs6006602 | SAMM50 | C | T | 1.266 | 4.210E-39 |
| 22 | rs4823109 | SAMM50 | C | T | 1.391 | 3.069E-41 |
| 22 | rs2281296 | SAMM50 | G | A | 0.839 | 4.571E-16 |
| 22 | rs6006473 | SAMM50 | C | T | 1.263 | 2.020E-38 |
| 22 | rs2235771 | SAMM50 | A | G | 0.870 | 1.349E-14 |
| 22 | rs67450864 | SAMM50 | C | T | 1.296 | 1.344E-44 |
| 22 | rs5764053 | SAMM50 | T | C | 0.888 | 1.179E-10 |
| 22 | rs738409 | PNPLA3 | C | G | 1.513 | 4.847E-88 |
| 22 | rs2294915 | PNPLA3 | C | T | 1.480 | 3.060E-82 |
| 22 | rs2073079 | SAMM50 | A | G | 1.288 | 1.096E-29 |
| 22 | rs738407 | PNPLA3 | T | C | 1.194 | 3.678E-21 |
| 22 | rs3788603 | SAMM50 | T | A | 0.836 | 1.217E-16 |
| 22 | rs139052 | PNPLA3 | C | A | 0.835 | 7.476E-18 |
| 22 | rs1883350 | PNPLA3 | T | C | 1.264 | 1.194E-33 |
| 22 | rs4823182 | SAMM50 | A | G | 1.297 | 7.116E-45 |
| 22 | rs5764434 | SAMM50 | C | T | 0.889 | 6.056E-11 |
| 22 | rs5764047 | SAMM50 | G | A | 0.836 | 1.082E-16 |
| 22 | rs4823173 | PNPLA3 | G | A | 1.437 | 2.799E-54 |
| 22 | rs738408 | PNPLA3 | C | T | 1.514 | 3.388E-88 |
| 22 | rs13054885 |  | G | A | 1.424 | 6.273E-53 |
| 22 | rs56219234 | SAMM50 | G | T | 1.231 | 7.342E-27 |
| 22 | rs16991236 | SAMM50 | A | G | 1.346 | 1.571E-19 |
| 22 | 22:44325479_CT_C | PNPLA3 | CT | C | 1.420 | 1.021E-51 |

* Linear closest gene is annotated by 3DSNP v1.0 (https://www.omic.tech/3dsnp/);

Abbreviations: SNPs, single nucleotide polymorphisms; LASSO, Least Absolute Shrinkage and Selection Operator; SE, standard error.

**Table S3. Mean performance metrics of single classifiers in the training set across 5-fold cross-validation.**

| Classifier | AUC | Accuracy | Sensitivity | Specificity |
| --- | --- | --- | --- | --- |
| AdaBoost | 0.834(0.009) | 0.752(0.011) | 0.767(0.011) | 0.745(0.019) |
| Support Vector Machine | 0.837(0.009) | 0.750(0.009) | 0.780(0.037) | 0.736(0.028) |
| Kneighbors | 0.650(0.005) | 0.606(0.033) | 0.613(0.100) | 0.603(0.096) |
| LightGBM | 0.841(0.007) | 0.760(0.006) | 0.766(0.021) | 0.757(0.013) |
| Logistic Regression | 0.837(0.008) | 0.759(0.006) | 0.755(0.029) | 0.761(0.019) |
| Random Forest | 0.828(0.007) | 0.745(0.010) | 0.766(0.030) | 0.735(0.027) |
| XGBoost | 0.839(0.008) | 0.758(0.007) | 0.764(0.029) | 0.756(0.021) |

Values are mean (SD) across 5 folds. Performance metrics for models are seven single classifiers;

Rows correspond to the model being tested;

Abbreviation: AUC, area under the receiver operating characteristic curve; SNP, single nucleotide polymorphism;

**Table S4.** **Comparison of clinical characteristics between low- and high-risk groups for NAFLD in training set.**

| Characteristic | Total (n = 29192) | Low-risk group  (n = 1,6550) | High-risk group  (n = 12,642) | *P* |
| --- | --- | --- | --- | --- |
| Age, years | 56 (49, 61) | 55 (48, 61) | 57 (51, 62) | < 0.001 |
| Sex, n (%) |  |  |  | < 0.001 |
| Female | 15,682 (53.72) | 10,055 (60.76) | 5,627 (44.51) |  |
| Male | 13,510 (46.28) | 6,495 (39.24) | 7,015 (55.49) |  |
| BMI, kg/m^2^ | 26.26 (23.77, 29.34) | 24.32 (22.58, 26.09) | 29.61 (27.35, 32.62) | < 0.001 |
| TG, mmol/L | 1.43 (1, 2.08) | 1.13 (0.86, 1.52) | 1.99 (1.47, 2.75) | < 0.001 |
| LDL-C, mmol/L | 3.51 (2.96, 4.10) | 3.47 (2.97, 4.03) | 3.57 (2.95, 4.19) | < 0.001 |
| CHO, mmol/L | 5.63 (4.93, 6.39) | 5.66 (5.01, 6.36) | 5.58 (4.80, 6.41) | < 0.001 |
| HDL-C, mmol/L | 1.41 (1.17, 1.68) | 1.58 (1.35, 1.83) | 1.21 (1.04, 1.40) | < 0.001 |
| FPG, mmol/L | 4.89 (4.57, 5.25) | 4.84 (4.53, 5.15) | 4.98 (4.62, 5.44) | < 0.001 |
| ALT, U/L | 20.02 (15.26, 27.65) | 16.95 (13.65, 21.23) | 26.82 (20.09, 36.83) | < 0.001 |
| AST, U/L | 24.4 (20.9, 28.9) | 23.1 (20.1, 26.6) | 26.6 (22.4, 32.5) | < 0.001 |
| ISMLD | 0.20 (0.10, 0.51) | 0.11 (0.08, 0.16) | 0.58 (0.38, 0.79) | < 0.001 |
| Smoking status, n (%) |  |  |  | < 0.001 |
| Smoker | 8,386 (28.73) | 4,631 (27.98) | 3,755 (29.70) |  |
| Non-smoker | 12,191 (41.76) | 7,681 (46.41) | 4,510 (35.67) |  |
| NA | 8,615 (29.51) | 4,238 (25.61) | 4,377 (34.62) |  |
| Alcohol consumption, n (%) | |  |  | < 0.001 |
| Moderate | 13,150 (45.05) | 8,410 (50.82) | 4,740 (37.49) |  |
| Never/mild | 7,427 (25.44) | 3,902 (23.58) | 3,525 (27.88) |  |
| NA | 8,615 (29.51) | 4,238 (25.61) | 4,377 (34.62) |  |
| Physical activity, n (%) | |  |  | < 0.001 |
| Inactive | 9,386 (32.15) | 5,111 (30.88) | 4,275 (33.82) |  |
| Active | 1,1191 (38.34) | 7,201 (43.51) | 3,990 (31.56) |  |
| NA | 8,615 (29.51) | 4,238 (25.61) | 4,377 (34.62) |  |
| Diet, n (%) ^a^ | |  |  | < 0.001 |
| Unhealthy | 7,893 (27.04) | 4,303 (26.00) | 3,590 (28.40) |  |
| Healthy | 12,684 (43.45) | 8,009 (48.39) | 4,675 (36.98) |  |
| NA | 8,615 (29.51) | 4,238 (25.61) | 4,377 (34.62) |  |
| Lifestyle, n (%) ^b^ |  |  |  | < 0.001 |
| Unfavorable | 5,656 (19.38) | 3,079 (18.60) | 2,577 (20.38) |  |
| Intermediate | 7,506 (25.71) | 4,513 (27.27) | 2,993 (23.68) |  |
| Favorable | 7,415 (25.40) | 4,720 (28.52) | 2,695 (21.32) |  |
| NA | 8,615 (29.51) | 4,238 (25.61) | 4,377 (34.62) |  |

^a^ Diet pattern included seven dietary components: fruits, vegetables, whole grains, refined grains, fish, unprocessed meat, and processed meat;

^b^ Lifestyle index was created by four healthy lifestyle factors: never/moderate alcohol consumption, no smoking, regular physical activity, and a healthy diet. Participants were categorized into three groups according to the number of healthy lifestyle factors: (1) unfavorable (0 or 1), (2) intermediate (any 2), and (3) favorable (3 or 4); Abbreviations: NAFLD, nonalcoholic fatty liver disease; BMI, Body mass index; TG, Triglyceride; CHO, Cholesterol; LDL-C, Low-density lipoprotein-cholesterol; HDL-C, High-density lipoprotein-cholesterol; Glu, blood glucose; ALT, alanine aminotransferase; AST, aspartate aminotransferase; ISNLD, in-silico score for NAFLD.

**Table S5. Comparison of clinical characteristics between low- and high-risk groups for NAFLD in internal test set.**

| Characteristic | Total (n = 7,298) | Low-risk group  (n = 4,126) | Hign-risk group  (n = 3,172) | *P* |
| --- | --- | --- | --- | --- |
| Age, years | 56 (50, 61) | 55 (49, 61) | 57 (50, 62) | < 0.001 |
| Sex, n (%) |  |  |  | < 0.001 |
| Female | 3,844 (52.67) | 2,465 (59.74) | 1,379 (43.47) |  |
| Male | 3,454 (47.33) | 1,661 (40.26) | 1,793 (56.53) |  |
| BMI, kg/m^2^ | 26.31 (23.73, 29.43) | 24.26 (22.55, 26.10) | 29.66 (27.33, 32.79) | < 0.001 |
| TG, mmol/L | 1.41 (1.01, 2.07) | 1.13 (0.86, 1.50) | 1.99 (1.45, 2.76) | < 0.001 |
| LDL-C, mmol/L | 3.50 (2.96, 4.09) | 3.46 (2.98, 4.01) | 3.54 (2.93, 4.18) | 0.003 |
| CHO, mmol/L | 5.62 (4.92, 6.37) | 5.66 (5.02, 6.36) | 5.56 (4.79, 6.38) | < 0.001 |
| HDL-C, mmol/L | 1.41 (1.18, 1.68) | 1.59 (1.35, 1.84) | 1.22 (1.03, 1.41) | < 0.001 |
| FPG, mmol/L | 4.89 (4.57, 5.27) | 4.84 (4.53, 5.16) | 4.99 (4.63, 5.44) | < 0.001 |
| ALT, U/L | 19.98 (15.22, 27.54) | 16.9 (13.52, 21.04) | 26.84 (20.05, 36.78) | < 0.001 |
| AST, U/L | 24.3 (21.0, 28.9) | 22.9 (20.0, 26.6) | 26.45 (22.7, 32.5) | < 0.001 |
| ISNLD | 0.20 (0.10, 0.52) | 0.10 (0.08, 0.15) | 0.58 (0.38, 0.78) | < 0.001 |
| Smoking status, n (%) | |  |  | < 0.001 |
| Smoker | 2,088 (28.61) | 1,148 (27.82) | 940 (29.63) |  |
| Non-smoker | 3,049 (41.78) | 1,885 (45.69) | 1,164 (36.70) |  |
| NA | 2,161 (29.61) | 1,093 (26.49) | 1,068 (33.67) |  |
| Alcohol consumption, n (%) | |  |  | < 0.001 |
| Moderate | 3,219 (44.11) | 2,066 (50.07) | 1,153 (36.35) |  |
| Never/mild | 1,918 (26.28) | 967 (23.44) | 951 (29.98) |  |
| NA | 2,161 (29.61) | 1,093 (26.49) | 1,068 (33.67) |  |
| Physical activity, n (%) | |  |  | < 0.001 |
| Inactive | 2,353 (32.24) | 1,289 (31.24) | 1,064 (33.54) |  |
| Active | 2,784 (38.15) | 1,744 (42.27) | 1,040 (32.79) |  |
| NA | 2,161 (29.61) | 1,093 (26.49) | 1,068 (33.67) |  |
| Diet, n (%) ^a^ | |  |  | < 0.001 |
| Unhealthy | 1,995 (27.34) | 1,093 (26.49) | 902 (28.44) |  |
| Healthy | 3,142 (43.05) | 1,940 (47.02) | 1,202 (37.89) |  |
| NA | 2,161 (29.61) | 1,093 (26.49) | 1,068 (33.67) |  |
| Lifestyle, n (%) ^b^ |  |  |  | < 0.001 |
| Unfavorable | 1,401 (19.20) | 7,60 (18.42) | 641 (20.21) |  |
| Intermediate | 1,864 (25.54) | 1,156 (28.02) | 708 (22.32) |  |
| Favorable | 1,872 (25.65) | 1,117 (27.07) | 755 (23.80) |  |
| NA | 2,161 (29.61) | 1,093 (26.49) | 1,068 (33.67) |  |

^a^ Diet pattern included seven dietary components: fruits, vegetables, whole grains, refined grains, fish, unprocessed meat, and processed meat;

^b^ Lifestyle index was created by four healthy lifestyle factors: never/moderate alcohol consumption, no smoking, regular physical activity, and a healthy diet. Participants were categorized into three groups according to the number of healthy lifestyle factors: (1) unfavorable (0 or 1), (2) intermediate (any 2), and (3) favorable (3 or 4); Abbreviations: NAFLD, nonalcoholic fatty liver disease; BMI, Body mass index; TG, Triglyceride; CHO, Cholesterol; LDL-C, Low-density lipoprotein-cholesterol; HDL-C, High-density lipoprotein-cholesterol; Glu, blood glucose; ALT, alanine aminotransferase; AST, aspartate aminotransferase; ISNLD, in-silico score for NAFLD.

**Table S6. Comparison of clinical characteristics between low- and high-risk groups for NAFLD in external test set.**

| Characteristic | Total  (n = 9,007) | Low-risk group  (n = 6,407) | High-risk group  (n = 2,600) | *P* |
| --- | --- | --- | --- | --- |
| Age, years | 36 (30, 45) | 34 (29, 44) | 39 (32, 48) | < 0.001 |
| Sex, n (%) |  |  |  | < 0.001 |
| Female | 4,838 (53.71) | 4,184 (65.3) | 654 (25.15) |  |
| Male | 4,169 (46.29) | 2,223 (34.7) | 1,946 (74.85) |  |
| BMI, kg/m^2^ | 22.63 (20.48, 25.06) | 21.5 (19.84, 23.33) | 25.97 (24.16, 27.84) | < 0.001 |
| TG, mmol/L | 1.09 (0.79, 1.62) | 0.94 (0.73, 1.25) | 1.81 (1.29, 2.54) | < 0.001 |
| LDL-C, mmol/L | 3.11 (2.65, 3.63) | 3.01 (2.57, 3.50) | 3.39 (2.92, 3.90) | < 0.001 |
| CHO, mmol/L | 5.02 (4.40, 5.70) | 4.93 (4.34, 5.59) | 5.29 (4.6, 5.95) | < 0.001 |
| HDL-C, mmol/L | 1.36 (1.18, 1.58) | 1.45 (1.27, 1.64) | 1.17 (1.04, 1.32) | < 0.001 |
| FPG, mmol/L | 4.77 (4.50, 5.08) | 4.72 (4.46, 4.99) | 4.95 (4.62, 5.34) | < 0.001 |
| ALT, U/L | 16 (11, 25) | 14 (10, 19) | 29 (20, 43) | < 0.001 |
| AST, U/L | 18 (15, 22) | 17 (14, 20) | 21.5 (18, 27) | < 0.001 |
| ISNLD | 0.14 (0.09, 0.29) | 0.10 (0.08, 0.15) | 0.49 (0.34, 0.70) | < 0.001 |

Abbreviations: NAFLD, nonalcoholic fatty liver disease; BMI, Body mass index; TG, Triglyceride; CHO, Cholesterol; LDL-C, Low-density lipoprotein-cholesterol; HDL-C, High-density lipoprotein-cholesterol; Glu, blood glucose; ALT, alanine aminotransferase; AST, aspartate aminotransferase; ISNLD, in-silico score for NAFLD.

**Table S7. The associations of ISNLD with metabolism-related outcomes in the high-risk group for NAFLD of training set.**

| Outcome | Model1 | | Model2 | | Model3 | |
| --- | --- | --- | --- | --- | --- | --- |
|  | HR (95% CI) | *P*-value | HR (95% CI) | *P*-value | HR (95% CI) | *P*-value |
| Hypertension | 5.14  (3.42, 7.73) | <0.001 | 3.85  (2.30, 6.46) | <0.001 | 4.25  (2.54, 7.10) | <0.001 |
| DmT2 | 32.56  (18.66, 56.80) | <0.001 | 24.50  (11.75, 51.09) | <0.001 | 28.87  (13.89, 60.01) | <0.001 |
| CKD | 3.88  (2.38, 6.32) | <0.001 | 3.21  (1.66, 6.20) | 0.001 | 3.52  (1.83, 6.78) | <0.001 |
| CAD | 4.51  (2.89, 7.02) | <0.001 | 3.77  (2.16, 6.57) | <0.001 | 3.95  (2.27, 6.87) | <0.001 |
| HF | 5.81  (3.22, 10.49) | <0.001 | 4.51  (2.03, 9.98) | <0.001 | 5.09  (2.31, 11.22) | <0.001 |
| AF | 2.61  (1.63, 4.16) | <0.001 | 2.63  (1.43, 4.86) | 0.002 | 2.78  (1.51, 5.10) | 0.001 |
| Stroke | 6.18  (2.92, 13.11) | <0.001 | 2.63  (1.02, 6.77) | 0.045 | 2.77  (1.08, 7.09) | 0.033 |
| All-cause death | 8.28  (5.59, 12.26) | <0.001 | 7.40  (4.45, 12.31) | <0.001 | 8.38  (5.06, 13.88) | <0.001 |

ISNLD, in-silico score for NAFLD; CAD, coronary artery disease; HF, Heart failure; CKD, chronic kidney disease; DmT2, Type 2 diabetes; AF, atrial fibrillation/atrial flutter; HR, Hazard ratio; CI, confidence interval;

Model 1 was unadjusted;

Model 2 was adjusted for smoking status, alcohol consumption, physical activity, and diet; Model 3 was adjusted for lifestyle; Participants were categorized into three groups according to the number of healthy lifestyle factors: (1) unfavorable (0 or 1 healthy lifestyle factors), (2) intermediate (2 factors), and (3) favorable (3 or 4 factors);

Low risk group was set as reference.

**Table S8.** **The associations of ISNLD groups with metabolism-related outcomes in the high-risk group for NAFLD of training set.**

| Outcome | ISMLD  Group | Model 1 | | Model 2 | | Model 3 | |
| --- | --- | --- | --- | --- | --- | --- | --- |
|  |  | HR (95% CI) | *P*-value | HR (95% CI) | *P*-value | HR (95% CI) | *P*-value |
| Hypertension | Intermediate risk | 1.52 (1.21, 1.92) | <0.001 | 1.25 (0.95, 1.65) | 0.118 | 1.27 (0.96, 1.68) | 0.091 |
|  | High risk | **2.70 (2.10, 3.47)** | **<0.001** | **2.34 (1.73, 3.18)** | **<0.001** | **2.45 (1.81, 3.32)** | **<0.001** |
| DmT2 | Intermediate risk | 3.47 (2.26, 5.33) | <0.001 | 3.93 (2.24, 6.88) | <0.001 | 3.91 (2.23, 6.86) | <0.001 |
|  | High risk | **8.94 (5.82, 13.71)** | **<0.001** | **8.74 (4.95, 15.44)** | **<0.001** | **8.93 (5.06, 15.74)** | **<0.001** |
| CKD | Intermediate risk | 1.42 (1.05, 1.91) | 0.022 | 1.41 (0.96, 2.08) | 0.082 | 1.42 (0.97, 2.10) | 0.074 |
|  | High risk | **2.11 (1.55, 2.88)** | **<0.001** | **2.01 (1.33, 3.04)** | **0.001** | **2.08 (1.38, 3.14)** | **<0.001** |
| CAD | Intermediate risk | 1.41 (1.07, 1.84) | 0.014 | 1.29 (0.94, 1.78) | 0.120 | 1.29 (0.93, 1.78) | 0.122 |
|  | High risk | **2.35 (1.78, 3.10)** | **<0.001** | **2.20 (1.57, 3.08)** | **<0.001** | **2.19 (1.57, 3.06)** | **<0.001** |
| HF | Intermediate risk | 1.41 (0.98, 2.03) | 0.065 | 1.39 (0.87, 2.23) | 0.167 | 1.41 (0.88, 2.25) | 0.154 |
|  | High risk | **2.41 (1.67, 3.49)** | **<0.001** | **2.15 (1.32, 3.52)** | **0.002** | **2.25 (1.38, 3.66)** | **0.001** |
| AF | Intermediate risk | 1.21 (0.92, 1.60) | 0.171 | 1.26 (0.89, 1.79) | 0.194 | 1.26 (0.89, 1.78) | 0.200 |
|  | High risk | **1.79 (1.34, 2.38)** | **<0.001** | **1.91 (1.32, 2.77)** | **0.001** | **1.91 (1.32, 2.76)** | **0.001** |
| Stroke | Intermediate risk | 1.10 (0.70, 1.73) | 0.673 | 0.95 (0.56, 1.61) | 0.854 | 0.94 (0.56, 1.58) | 0.811 |
|  | High risk | **2.43 (1.56, 3.79)** | **<0.001** | **1.74 (1.01, 3.00)** | **0.046** | **1.72 (1.00, 2.95)** | **0.049** |
| All-cause death | Intermediate risk | 1.67 (1.29, 2.16) | <0.001 | 1.86 (1.34, 2.59) | <0.001 | 1.85 (1.33, 2.57) | <0.001 |
|  | High risk | **3.22 (2.49, 4.15)** | **<0.001** | **3.34 (2.39, 4.66)** | **<0.001** | **3.42 (2.46, 4.77)** | **<0.001** |

ISNLD, in-silico score for NAFLD; CAD, coronary artery disease; HF, Heart failure; CKD, chronic kidney disease; DmT2, Type 2 diabetes; AF, atrial fibrillation/atrial flutter; HR, Hazard ratio; CI, confidence interval;

Model 1 was unadjusted;

Model 2 was adjusted for smoking status, alcohol consumption, physical activity, and diet; Model 3 was adjusted for lifestyle; Participants were categorized into three groups according to the number of healthy lifestyle factors: (1) unfavorable (0 or 1 healthy lifestyle factors), (2) intermediate (2 factors), and (3) favorable (3 or 4 factors);

Low risk group was set as reference.

**Table S9**. **The associations of ISNLD with metabolism-related outcomes in the high-risk group for NAFLD of internal test set.**

| Outcome | Model1 | | Model2 | | Model3 | |
| --- | --- | --- | --- | --- | --- | --- |
|  | HR (95% CI) | *P*-value | HR (95% CI) | *P*-value | HR (95% CI) | *P*-value |
| Hypertension | 2.24  (1.00, 5.02) | 0.050 | 2.42  (0.89, 6.63) | 0.085 | 2.53  (0.92, 6.90) | 0.071 |
| DmT2 | 27.64  (9.41, 81.19) | <0.001 | 23.78  (5.28, 107.07) | <0.001 | 25.98  (5.71, 118.25) | <0.001 |
| CKD | 5.83  (2.10, 16.18) | 0.001 | 7.25  (2.02, 25.97) | 0.002 | 8.70  (2.40, 31.45) | 0.001 |
| CAD | 5.85  (2.54, 13.50) | <0.001 | 6.08  (2.16, 17.13) | 0.001 | 7.03  (2.49, 19.81) | <0.001 |
| HF | 2.83  (0.79, 10.10) | 0.109 | 3.52  (0.62, 19.89) | 0.155 | 3.22  (0.57, 18.13) | 0.185 |
| AF | 2.42  (0.93, 6.28) | 0.071 | 1.83  (0.53, 6.33) | 0.342 | 1.89  (0.54, 6.58) | 0.318 |
| Stroke | 1.48  (0.32, 6.90) | 0.617 | 2.25  (0.28, 18.41) | 0.449 | 2.23  (0.27, 18.18) | 0.455 |
| All-cause death | 5.60  (2.64, 11.88) | <0.001 | 6.91  (2.65, 18.04) | <0.001 | 7.89  (3.01, 20.66) | <0.001 |

ISNLD, in-silico score for NAFLD; CAD, coronary artery disease; HF, Heart failure; CKD, chronic kidney disease; DmT2, Type 2 diabetes; AF, atrial fibrillation/atrial flutter; HR, Hazard ratio; CI, confidence interval;

Model 1 was unadjusted;

Model 2 was adjusted for smoking status, alcohol consumption, physical activity, and diet; Model 3 was adjusted for lifestyle; Participants were categorized into three groups according to the number of healthy lifestyle factors: (1) unfavorable (0 or 1 healthy lifestyle factors), (2) intermediate (2 factors), and (3) favorable (3 or 4 factors);

Low risk group was set as reference.

**Table S10.** **The associations of ISNLD groups with metabolism-related outcomes in the high-risk group for NAFLD of internal test set.**

| Outcome | ISMLD  Group | Model 1 | | Model 2 | | Model 3 | |
| --- | --- | --- | --- | --- | --- | --- | --- |
|  |  | HR (95% CI) | *P*-value | HR (95% CI) | *P*-value | HR (95% CI) | *P*-value |
| SeLD | Intermediate risk | 3.40 (0.42, 27.64) | 0.253 | 2.67 (0.31, 22.88) | 0.371 | 2.73 (0.32, 23.38) | 0.359 |
|  | High risk | **9.57 (1.23, 74.71)** | **0.031** | **9.80 (1.23, 78.11)** | **0.031** | **10.16 (1.28, 80.54)** | **0.028** |
| Hypertension | Intermediate risk | 1.39 (0.91, 2.11) | 0.130 | 1.31 (0.78, 2.21) | 0.306 | 1.35 (0.80, 2.27) | 0.256 |
|  | High risk | **1.61 (0.98, 2.66)** | **0.062** | **1.76 (0.96, 3.23)** | **0.068** | **1.83 (1.00, 3.36)** | **0.051** |
| DmT2 | Intermediate risk | 1.56 (0.81, 2.99) | 0.185 | 1.14 (0.49, 2.66) | 0.765 | 1.15 (0.49, 2.69) | 0.745 |
|  | High risk | **4.54 (2.40, 8.58)** | **<0.001** | **3.37 (1.49, 7.63)** | **0.004** | **3.54 (1.56, 8.05)** | **0.002** |
| CKD | Intermediate risk | 1.24 (0.65, 2.35) | 0.512 | 1.44 (0.64, 3.23) | 0.373 | 1.48 (0.66, 3.32) | 0.340 |
|  | High risk | **2.74 (1.47, 5.14)** | **0.002** | **3.04 (1.37, 6.77)** | **0.006** | **3.41 (1.53, 7.58)** | **0.003** |
| CAD | Intermediate risk | 1.25 (0.75, 2.07) | 0.394 | 1.29 (0.69, 2.43) | 0.422 | 1.31 (0.70, 2.46) | 0.399 |
|  | High risk | **2.79 (1.69, 4.61)** | **<0.001** | **3.03 (1.62, 5.65)** | **<0.001** | **3.29 (1.77, 6.12)** | **<0.001** |
| HF | Intermediate risk | 1.69 (0.77, 3.71) | 0.190 | 1.24 (0.47, 3.26) | 0.664 | 1.17 (0.44, 3.08) | 0.753 |
|  | High risk | **1.92 (0.83, 4.44)** | **0.127** | **1.97 (0.70, 5.51)** | **0.197** | **1.86 (0.67, 5.18)** | **0.236** |
| AF | Intermediate risk | 1.30 (0.75, 2.24) | 0.348 | 1.36 (0.69, 2.66) | 0.377 | 1.31 (0.67, 2.57) | 0.431 |
|  | High risk | **1.48 (0.82, 2.67)** | **0.197** | **1.22 (0.56, 2.70)** | **0.615** | **1.25 (0.57, 2.73)** | **0.584** |
| Stroke | Intermediate risk | 0.92 (0.39, 2.18) | 0.857 | 1.07 (0.32, 3.57) | 0.909 | 1.08 (0.33, 3.60) | 0.895 |
|  | High risk | **1.43 (0.58, 3.50)** | **0.436** | **1.91 (0.55, 6.57)** | **0.307** | **1.88 (0.55, 6.50)** | **0.316** |
| All-cause death | Intermediate risk | 1.66 (1.02, 2.70) | 0.040 | 1.86 (1.00, 3.46) | 0.048 | 1.88 (1.02, 3.49) | 0.044 |
|  | High risk | **2.95 (1.81, 4.81)** | **<0.001** | **3.17 (1.69, 5.95)** | **<0.001** | **3.42 (1.83, 6.42)** | **<0.001** |

ISNLD, in-silico score for NAFLD; CAD, coronary artery disease; HF, Heart failure; CKD, chronic kidney disease; DmT2, Type 2 diabetes; AF, atrial fibrillation/atrial flutter; HR, Hazard ratio; CI, confidence interval;

Model 1 was unadjusted;

Model 2 was adjusted for smoking status, alcohol consumption, physical activity, and diet; Model 3 was adjusted for lifestyle; Participants were categorized into three groups according to the number of healthy lifestyle factors: (1) unfavorable (0 or 1 healthy lifestyle factors), (2) intermediate (2 factors), and (3) favorable (3 or 4 factors);

Low risk group was set as reference.

**Table S11. Definition of lifestyle factors in the UK Biobank.**

| Factors | Unhealthy level | Healthy level |
| --- | --- | --- |
| Alcohol consumption | women: ≥1 unit /day  men: ≥2 units /day  one unit is measured as 8 g (10 ml) ethanol in the U.K. | women: 0 and <1 units /day  men: 0 and <2 units /day  or never deinked  one unit is measured as 8 g (10 ml) ethanol in the U.K. |
| Smoking status | Smoking | Non-smoking |
| Physical activity | 0-149 min/week of moderate activity OR 0-74 min/week of vigorous activity OR 0-149 min/week of moderate and vigorous activity | ≥150 min/week of moderate activity OR ≥75 min/week of vigorous activity OR ≥150 min/week of moderate and vigorous activity |
| Diet | Intake of 0-3 healthy dietary components:  1. Fruits: ≥ 3 servings/day  2. Vegetables: ≥ 3 servings/day  3. Fish: ≥2 servings/week  4. Processed meats: ≤ 1 serving/week  5.Unprocessed red meats: ≤1.5 servings/week  6. Whole grains: ≥ 3servings/day  7. Refined grains: ≤1.5 servings/day | Intake of ≥ 4 healthy dietary components:  1. Fruits: ≥ 3 servings/day  2. Vegetables: ≥ 3 servings/day  3. Fish: ≥2 servings/week  4. Processed meats: ≤ 1 serving/week  5.Unprocessed red meats: ≤1.5 servings/week  6. Whole grains: ≥ 3servings/day  7. Refined grains: ≤1.5 servings/day |

**Table S12. Coding Algorithms for Defining diseases in the UK Biobank.**

| Diseases | ICD-10 code | ICD-9 code | Others |
| --- | --- | --- | --- |
| Non-alcoholic fatty liver disease | K740, K746, K758, K760 | 5715, 5718, 5719 | MRI-PDFF>5%;  Exclude individuals with liver disease other than Non-alcoholic fatty liver disease |
| Severe liver disease | C220, I850, I859, K703, K704, K721, K741, K742, K746, K766, K767, Z944 | 1550,4560,5308,4561,7476,4562A,5715,5712,571,570,5718,5713,5722,5719,5715,5723,5724, V427 |  |
| Type 2 Diabetes | E11 | 250, 2500, 2509, 6480, 7902 | **Non-cancer illness**: 1223 (type 2 diabetes) |
| Hypertension | I10, I11, I12, I13, I15, O10 | 401, 402, 403, 404, 405 | **Medication**: ACE inhibitors, AT-II antagonists, Beta-blockers, Calcium channel-blockers, Thiazide diuretics;  **Non-cancer illness**: 1065 (hypertension), 1072 (essential hypertension) |
| Chronic kidney disease | I120, I131, I132, N18, N180, N181, N182, N183, N184, N185, N188, N189 | 585, 5859 |  |
| Coronary artery disease | I21, I22, I23, I252, Z951 | 410, 412, 414 | **Non-cancer illness**: 1075 (heart attack/myocardial infarction);  **Operation**: 1070 (coronary angioplasty +/- stent), 1095 (cabg), 1523 (triple heart bypass) |
| Heart failure | I50, I110, I130, I132, Z941, T862 | 428 | **Non-cancer illness:** 1076 (heart failure/pulmonary odema);  **Operation:** 1098 (heart transplant) |
| Atrial fibrillation/Atrial flutter | I48 | 4273 |  |
| Stroke | I607, I600, I609, I619, I611, I614, I613, I615, I616, I629, I630, I632, I635, I678, I64X, I690, I612, I693, I606, I608, I618, I638, I60, I61, I601, I602, I603, I604, I64, I633 | 430,431,7670,432,4329,  4340,4341, |  |

*Self-report data in UK Biobank: Non-cancer illness, data-Field 20002; Treatment/Medication, data-Field 20003; Operation: data-Field 20004.
